# Aging and aging-related diseases: from molecular mechanisms to interventions and treatments

**DOI:** 10.1038/s41392-022-01251-0

**Published:** 2022-12-16

**Authors:** Jun Guo, Xiuqing Huang, Lin Dou, Mingjing Yan, Tao Shen, Weiqing Tang, Jian Li

**Affiliations:** The Key Laboratory of Geriatrics, Beijing Institute of Geriatrics, Institute of Geriatric Medicine, Chinese Academy of Medical Sciences, Beijing Hospital/National Center of Gerontology of National Health Commission, Beijing, 100730 China

**Keywords:** Cell biology

## Abstract

Aging is a gradual and irreversible pathophysiological process. It presents with declines in tissue and cell functions and significant increases in the risks of various aging-related diseases, including neurodegenerative diseases, cardiovascular diseases, metabolic diseases, musculoskeletal diseases, and immune system diseases. Although the development of modern medicine has promoted human health and greatly extended life expectancy, with the aging of society, a variety of chronic diseases have gradually become the most important causes of disability and death in elderly individuals. Current research on aging focuses on elucidating how various endogenous and exogenous stresses (such as genomic instability, telomere dysfunction, epigenetic alterations, loss of proteostasis, compromise of autophagy, mitochondrial dysfunction, cellular senescence, stem cell exhaustion, altered intercellular communication, deregulated nutrient sensing) participate in the regulation of aging. Furthermore, thorough research on the pathogenesis of aging to identify interventions that promote health and longevity (such as caloric restriction, microbiota transplantation, and nutritional intervention) and clinical treatment methods for aging-related diseases (depletion of senescent cells, stem cell therapy, antioxidative and anti-inflammatory treatments, and hormone replacement therapy) could decrease the incidence and development of aging-related diseases and in turn promote healthy aging and longevity.

## Introduction

**Fig. 1:**
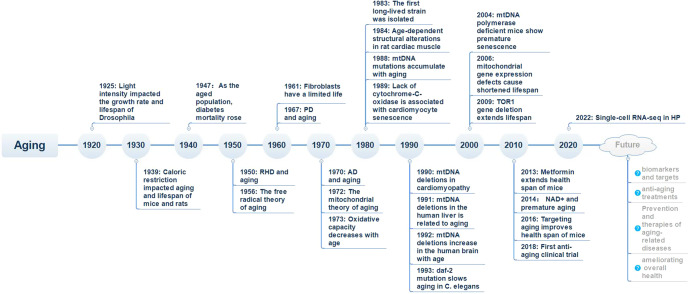
Timeline of research on aging and aging-related diseases. RHD rheumatic heart disease, PD Parkinson’s disease, AD Alzheimer’s disease, mtDNA mitochondrial DNA, NAD^+^ nicotinamide adenine dinucleotide, HP heterochronic parabiosis

Aging is a ubiquitous biological process that results in a progressive and irreversible decline in physical function across all organ systems that is induced by the accumulation of damage in response to a variety of stressors. Surprisingly, in 1925, research revealed that light intensity could impact the growth rate and lifespan of Drosophila.^1^ This finding attracted a considerable amount of research interest in the aging area and captured the curiosity and imagination of the public (Fig. 1). In addition, caloric restriction (CR) has also been found to impact aging, age-related pathologies, and longevity in mice and rats.^2^ These findings show that the plasticity of the aging process is critical for longevity. Approximately 30 years ago, the first long-lived strain was isolated from *C. elegans*, and this discovery ushered in a new era of aging research.^3^ For several decades, biologists have suggested that there may be an unappreciated but important link between aging and many chronic disorders in humans, and aging increases the risks of many common diseases,^4^ including diabetes,^5^ Alzheimer’s disease (AD),^6^ Parkinson’s disease (PD),^7^ cardiovascular disease,^8,9^ chronic obstructive pulmonary disease (COPD),^10^ osteoporosis (OP),^11^ and even osteoarthritis (OA).^12^ Moreover, many older patients have multiple comorbidities with advancing age, especially after the age of 60 years. These patients require combinations of different treatments to attain therapeutic effects in the long term.^13^ In addition, the existing therapeutic options for many age-related diseases likely affect each other.^14^ Since aging is one of the leading risk factors for most chronic diseases, it is anticipated that understanding the aging process will facilitate the identification of therapeutic targets for age-related diseases and the development of pharmacological agents suitable for approved clinical use in the future.^15^

Cellular senescence, with major effects on the maintenance of normal tissue homeostasis, as well as pathological conditions, is a principle causative factor in the organismal aging process and facilitates aging and aging-associated diseases. Cellular senescence is a gradual decline of the proliferation and differentiation ability as well as the physiological function of cells over time. In 1956, Denham Harman proposed the free radical theory of aging, arguing that degenerative changes during aging are mediated by the harmful effects of free radicals generated during normal cellular metabolism.^16^ In 1961, in the continuous culture of human diploid cells, the lifespan of fibroblasts was first found to be limited.^17^ Thus far, a number of molecular mechanisms that regulate aging have been discovered, such as telomere dysfunction,^18^ loss of proteostasis,^19^ mitochondrial dysfunction,^20^ stem cell exhaustion,^21^ and epigenetic alterations.^22^ The aging process is driven by a number of complex and important pathways, and many of those drivers are associated with chronic oxidative stress caused by elevated levels of reactive oxygen species (ROS).^23^

Here, we will focus on recent findings on the biological processes of aging, highlight the important roles of aging in multiple aging-related diseases in humans, and discuss efficient interventions and treatments.

## Molecular mechanisms of aging

**Fig. 2:**
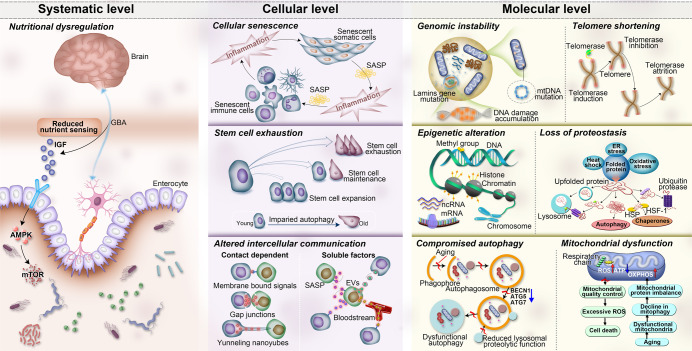
The ten hallmarks of aging are subdivided into three categories: molecular hallmarks (genomic instability, telomere dysfunction, epigenetic alterations, loss of proteostasis, compromise of autophagy, and mitochondrial dysfunction), cellular hallmarks (cellular senescence, stem cell exhaustion, and altered intercellular communication), and systemic alterations (deregulated nutrient sensing). AMPK protein kinase AMP-activated catalytic subunit alpha 1, ATG5: autophagy-related 5, ATG-7 autophagy-related 7, ATP adenosine triphosphate, BECN1 Beclin 1, ER endoplasmic reticulum stress, EVs extracellular vesicles, GBA gut–brain axis, HSF-1 heat shock factor-1, HSP heat shock protein, IGF insulin-like growth factor-1, mtDNA mitochondrial DNA, mRNA messenger RNA, mTOR mechanistic target of rapamycin kinase, ncRNA noncoding RNA, OXPHOS oxidative phosphorylation, Rb retinoblastoma, ROS, reactive oxygen species, SASP senescence-associated secretory phenotype

In 2013, López-Otín C et al. summarized aging in different organisms (especially mammals) as consisting of nine common features, including genomic instability, telomere attrition, epigenetic alteration, loss of proteostasis, deregulation of nutrient sensing, mitochondrial dysfunction, cellular senescence, stem cell exhaustion, and alteration of intercellular communication.^24^ Over the years, the distinction between compromised autophagy and impaired proteostasis has enabled members of the aging field to propose a compromise of autophagy as the 10th hallmark of aging. This new concept was proposed by the main authors (Linda Partridge and Guido Kroemer) of a 2013 *Cell* paper, in addition to other aging and autophagy researchers.^25^ In recent years, scientists have made important achievements in research on signaling pathways and molecular mechanisms that affect the aging process. Here, we analyze and summarize these new advances at three different levels: the molecular level (genomic instability, telomere dysfunction, epigenetic alterations, loss of proteostasis, compromise of autophagy, mitochondrial dysfunction), the cellular level (cellular senescence, stem cell exhaustion, and intercellular communication), and systemic alterations (deregulated nutrient sensing). This information may broaden the understanding of multiple molecular signaling networks involved in the aging processes of organisms (Fig. 2).

### Genomic instability

Genomic instability is an important cause of cellular senescence in many species.^26^ It leads to the deregulation of gene expression and consequently to impairment of cellular physiological aging, cessation of cell growth, and eventually cell death.^26^

#### Nuclear DNA damage accumulation

Endogenous DNA damage is deemed a major marker of genomic instability, especially DNA double-strand breaks.^27^ DNA damage activates the DNA damage response and cell cycle checkpoint pathways, such as the p53-p21 and p16INK4a-pRb pathways. Thus, the cell cycle is blocked to prevent the transmission of damaged genetic information to offspring cells.^28–31^ Tabula et al. performed single-cell RNA sequencing of more than 350,000 cells from male and female C57BL/6JN mice in six age groups ranging from 1 month (equivalent to human early childhood) to 30 months (equivalent to human centenarians).^32^ They analyzed changes in aging markers (including genomic instability) in 23 tissues and organs by Tabula Muris Senis, a tool capable of identifying aging-related changes in specific cell types.^32^ The researchers mapped the proportion of cells expressing each marker in all age groups and confirmed that the number of genomic mutations was positively associated with aging in all organs, with the tongue and bladder being the most affected.^32^ Analyses of centenarians have shown that long-lived individuals have fewer somatic and germ cell mutations than the general population, suggesting that they have more efficient DNA repair mechanisms to maintain genomic stability.^33,34^ Persistent foci of nuclear DNA damage in senescent cells are also known as DNA segments with chromatin alterations reinforcing senescence (DNA-SCARSs).^35^ DNA-SCARSs are dynamic structures that may regulate multiple aspects of senescence in cells, including growth arrest and the senescence-associated secretory phenotype (SASP).^35^ During cellular senescence, loss of the nuclear membrane protein Lamin B17,11,12 leads to impaired integrity of the nuclear envelope, causing chromatin fragment-containing nuclear membranes to enter the cytoplasm as cytoplasmic chromatin fragments (CCF).^36^ Further studies revealed that in senescent cells, CCF is recognized by cyclic guanosine monophosphate-adenosine monophosphate synthase (cGAS), producing a second messenger cyclic GMP-AMP (cGAMP) that activates the stimulator of interferon genes (STING), which mediates DNA damage-induced SASP and tissue inflammatory damage in vivo.^36^

#### Mitochondrial DNA (mtDNA)

Unlike nuclear DNA, mtDNA contains only exons and lacks histone protection and an effective gene repair system. These features make mtDNA more susceptible to mutations than nuclear DNA.^37^ In addition, considering the role of mitochondria as cellular powerhouses, some high-energy electron leakage may occur in the respiratory chain, which constitutes a major source of oxidative stress and subsequent elevated mutation rates in mtDNA.^38^ Decreased mtDNA copy numbers and increased numbers of mtDNA mutations are observed in various tissues in aging organisms.^39,40^ Oxidative stress also leads to the release of mtDNA into the cytoplasm.^40^ The released mtDNA can bind to cGAS and participate in cellular senescence by activating STING. STING is closely associated with the SASP.^41,42^ Moreover, mtDNA is exported extracellularly and detected as circulating cell-free mtDNA in extracellular fluid and cerebrospinal fluid.^43^ Circulating cell-free mtDNA is a novel signal for mitochondrial communication between distal tissues and has been associated with neurological disorders and systemic inflammation.^43^ A recent study found that a deficiency of melatonin, an endogenous free radical scavenger that decreases with age and neurodegeneration, impairs mitochondrial homeostasis, leading to mtDNA release and activation of the cGAS/STING/IRF3 pathway, which stimulates inflammatory cytokine production.^44^ By supplementing with exogenous melatonin, mtDNA release, cGAS activation, and inflammatory responses were significantly ameliorated in Huntington’s disease mice.^44^

#### Nuclear architecture

Alterations in the function of the nuclear lamina may be drivers of normal aging.^45^ The products of the lamin A/C (LMNA) genes, mainly lamin A and C, are key components of the nuclear lamina and are essential for maintaining proper nuclear structure.^45^ Hundreds of LMNA gene mutations have been identified in multiple degenerative-related diseases, including neuropathies, muscular dystrophy, lipodystrophy, and Hutchinson-Gilford syndrome.^45^ The absence of Lamin B1 is also a well-recognized feature of cellular senescence, and Sebastian Jessberger et al. showed that the levels of lamin B1 decrease with age in humans.^46^ After the levels of lamin B1 are increased in aging mice, neural stem cell division is improved, and the number of new neurons increases.^46^

#### Endogenous cytoplasmic DNA

Endogenous cellular DNA is a contributing factor to aseptic inflammation, which occurs in the absence of pathogenic infection. Additionally, it is associated with the development of many chronic diseases, including cardiovascular disease and neurodegenerative disorders.^47^ Aseptic inflammation is an important marker of immune senescence and has been reported to be triggered by endogenous cellular DNA.^47,48^ A recent study identified significant increases in cytoplasmic DNA levels in the CD^4+^ T cells of elderly humans and mice and revealed that this increase was accompanied by elevated expression of the KU complex (heterodimeric DNA-targeting component including 70 and 83 kDa subunits, Ku70 and Ku80).^48,49^ Further studies revealed that KU-mediated DNA sensing promotes the activation of the AKT and mammalian target of rapamycin (mTOR) pathways. This, in turn, stimulates the proliferation and activation of CD^4+^ T cells, thereby enhancing aging-associated autoimmunity.^48^

#### Junk DNA

Almost 50% of the human genome is made up of repetitive DNA that does not encode proteins.^50^ These DNA sequences are often considered to be “junk DNA” in the human genome.^50^ Research has shown that one of the junk DNA sequences, VNTR2-1, actually functions to enhance telomerase gene activity and that the telomerase gene is more active in people with longer VNTR2-1 sequences.^51^ Notably, however, a shorter sequence does not necessarily mean a shorter lifespan. Instead, it implies lower telomerase gene activity and a shorter telomere length, which may reduce the likelihood of developing cancer.^51^ This finding suggests that junk DNA also contributes to the genetic diversity of aging.^51^

### Telomere dysfunction

Telomeric DNA shortens with increasing numbers of cell divisions, and when it shortens to the Hayflick limit, telomere dysfunction causes a DNA damage response. This, in turn, induces cell cycle arrest and proinflammatory factor expression, ultimately leading to organismal aging.^52^ Telomere length is controlled by telomerase activity, and when telomerase activity is enhanced and the integrity of chromosomes is improved, the lifespan of an organism is prolonged.^52^

#### Telomeres

A telomere is a nuclear protein structure formed by telomeric DNA and binding proteins.^52^ In mammals, telomeres are formed by a highly conserved hexameric tandemly repeated DNA sequence (TTAGGG).^52^ They are located at the end of each chromosome arm and are designed to maintain genomic stability.^53^ The association between telomeric sequences and the shelterin complex leads to a stalled replication fork.^53^ However, once telomere shortening exceeds a critical level, the proteins forming the shelterin complex can no longer associate with telomeric sequences and interact with capping chromosome ends.^52^ Thus, a major limiting factor for telomere function is telomere length. Numerous studies have confirmed that telomere length is inversely proportional to age.^54–58^ However, notably, telomere length is highly variable in individuals of the same or similar ages.^59^

#### Telomerase

Telomerase activity is highest in human embryonic tissues and decreases progressively with age.^60^ In the classic view, telomerase protects certain frequently dividing cells in normal tissues, such as embryonic cells, sperm cells, adult stem cells, and immune cells, but it is inactive in other cells.^61^ Research from the University of Maryland has revealed that telomerase is reactivated in normal adult cells at critical moments during the aging process to mitigate the effects of aging, allowing cells to gradually die.^61^ The same research has also revealed that human cells express telomerase as they approach the telomere critical length.^61^ In addition, human skin cells that fail to express telomerase reach the telomere critical length more quickly and show more DNA damage than those that successfully express telomerase.^61^ The intervening role of telomerase in aging-related diseases has been demonstrated.^62–64^ In a mouse model of Hutchinson-Gilford progeria syndrome (HGPS), overexpression of telomerase mRNA reduced markers of inflammation and DNA damage in endothelial cells of various organs and prolonged the lifespan of HGPS mice.^62^ Aging is one of the independent determinants of hepatic telomere decompensation, and in hepatocellular carcinoma (HCC) cell lines, silencing telomerase reverse transcriptase inhibits cell proliferation by shortening telomeres, reducing DNA damage, and inducing apoptosis.^63^ However, overexpression of telomerase occurs in ~90% of cancers, making scientists wary of telomerase-related antiaging therapies.^65^

### Epigenetic alterations

Epigenetics refers to changes in phenotype or gene expression caused by mechanisms other than changes in DNA sequence.^66^ Epigenetic mechanisms include DNA methylation, histone modifications, chromatin remodeling, and transcriptional alteration by noncoding RNAs (ncRNAs).^66^

#### DNA methylation

DNA methylation regulates gene expression by recruiting proteins involved in gene repression or by inhibiting the binding of transcription factors to DNA.^66^ Methylation at the fifth position of cytosine (5mC) in DNA is a main epigenetic modification in mammals.^67^ 5-Hydroxymethylcytosine (5hmC) is a stable DNA base modification that results from 5-methylcytosine via the actions of the ten-eleven translocation protein family.^68^ Both of these modifications function as epigenetic markers.^67,68^ Compared to quiescent or proliferating cells, senescent cells exhibit fewer cytosine modifications.^68^ A recent study has reported that 5mC deamination and oxidative damage are major contributors to somatic mutagenesis, which scales with lifespan across mammals.^69^ It is well-established that DNA methylation changes with age in a process termed “epigenetic drift”.^70^ DNA methylation decreases during aging, mainly in the regions comprising heterochromatin repeats, and hypermethylation occurs in the regions of promoter CpGs.^66^ In 2013, Steve Horvath noted the correlation between methylation and age and thus described an epigenetic clock. This clock measures our biological age and predicts our lifespan.^71^ In 2018, Levine developed a second epigenetic clock, the “DNAm PhenoAge”. This clock confirmed the intrinsic links between the epigenetic clock and some aging mechanisms, such as the activation of proinflammatory and interferon pathways, transcriptional and translational mechanisms, and the DNA damage response.^72^ A growing number of studies have confirmed that aging-related changes can be determined using DNA methylation.^73–77^ For instance, Shireby et al. showed that epigenetic clocks can provide insight into the aging process of the human brain and predict the risk of dementia.^73^ In addition, the biological clocks of people with fatty liver move more quickly than normal, while those of centenarians move more slowly.^74^ In a study that included “Diet, Physical Activity, and Mammography” (DAMA), improved dietary habits slows the DNA mGrimAge clock and promotes healthy aging in the women tested.^75^ Blood-DNA methylation is sensitive to physiological changes that occur in multiple organ systems, and Belsky et al. identified a blood-DNA-methylation measure, DunedinPoAm, a method that estimates the rate of aging in subjects in the years prior to the measurement.^77^ They found that DunedinPoAm was associated with an increased risk of chronic disease incidence and mortality in older men.^77^ Moreover, adolescents raised in families with lower socioeconomic status exhibited faster DunedinPoAm, which often predicted a shorter healthy lifespan.^77^

#### Histone modification

Histone modifications include several different types: acetylation, methylation, phosphorylation, ubiquitination, glycosylation, ADP-ribosylation, deamination, and proline isomerization.^78^ Among them, acetylation and methylation are the two most characteristic modifications associated with senescence.^78^ In the presence of histone methyltransferases or histone demethylases, the methylation levels of histones are altered, and these alterations participate in transcriptional activation or transcriptional repression.^79^ In general, methylation at lysine 4 of histone 3 (H3K4), H3K36, and H3K79 promotes transcriptional activation, while methylation at H3K27 and H4K20 causes transcriptional repression.^79^ In contrast, trimethylation, dimethylation, and single methylation at H3K9 (H3K9me3, H3K9me2, and H3K9me, respectively) have different effects, and their functions depend on the methylated sites and methylation types.^80^ Studies have confirmed that histone methylation is altered during aging.^81^ For example, models of premature aging diseases, such as Hutchinson-Gilford syndrome and Werner syndrome, exhibit loss of heterochromatin and a decrease in H3K9me3 or SUV39H (an H3K9me3 histone methyltransferase).^79,82^ Histone acetyltransferases or histone deacetylases catalyze histone acetylation or deacetylation reactions. Histone acetyltransferases are usually transcriptionally activators, and histone deacetylases exert transcriptional repression functions.^81^ Histone acetyltransferases and histone deacetylases play key roles in longevity.^83^ For example, deletion of the histone acetyltransferase gene GCN5 shortens the replicative lifespan of yeast.^81^ Given the sophisticated links between aging and histone modifications, reversal of aging via intervention in histone modifications could be a potential therapeutic strategy.

#### Chromatin remodeling

Epigenetic studies have confirmed the widespread loss of histones and local and global remodeling of chromatin with age.^84,85^ For instance, the epigenomes of senescent cells show loss of chromatin rigidity, increased entropy, disorganization of the epigenome, reduced compartmentalization, convergent changes in genome-wide epigenetic signatures, and reduced polarity.^86^ Researchers have defined this phenomenon as convergent alteration of the epigenomic landscape during aging.^86^ Chromatin remodellers alter chromatin structure and nucleosome position through an ATP-dependent enzyme similar to helicase, enabling regulatory proteins to contact DNA.^87^ Liu et al. found that the Switch/sucrose non-fermentable (SWI2/SNF2) complex core structural domain contacts each other through two induced Brace helices, anchoring chromatin remodellers to fixed nucleosome positions and initiating substrates for remodeling reactions.^87^ Under senescence-induced mitochondrial stress, impaired tricarboxylic acid cycle leads to reduced acetyl coenzyme (acetyl-CoA) production, which induces nuclear accumulation of histone deacetylase and homeobox protein dve-1 and reduces histone acetylation and chromatin reorganization in *C. elegans*.^85^ Conversely, the addition of nutrients that promote acetyl-CoA production is sufficient to delay the lifespan of *C. elegans* after mitochondrial stress occurs.^85^ These findings provide new insights into chromatin remodeling. Chromatin accessibility is also a common feature of active regulatory elements, including enhancers, promoters, insulators, and chromatin-binding factors, to which transcription factors can be recruited by DNA-specific interactions.^88^ Aging cells present generally elevated chromatin accessibility, but the genome-wide profile differs depending on the stimulus.^35^ During human umbilical vein endothelial cell (HUVEC) senescence, chromatin accessibility, mainly referring to intergenic chromatin along with increased accessibility regions (IARs) or decreased accessibility regions (DARs), is redistributed.^89^ This process is mainly initiated by activating transcription factor 3 (ATF3) in the Jun proto-oncogene, AP-1transcription factor subunit (AP-1) transcription factor family, in which low DNA methylation enhances the binding affinity of AP-1, further increasing chromatin accessibility, facilitating chromatin rebuilding and driving the senescence program in HUVEC.^89^ In addition to structural abnormalities, numerical abnormalities such as aneuploidy and heteroploidy also contribute to aging.^90,91^ Numerical abnormalities, also known as aneuploidy, involve autosomes (chromosomes 1–22) as well as gonosomes (X or Y chromosomes).^91,92^ Unlike in young people, chromosomal deletions are significantly increased in cultured lymphocytes from the elderly.^92,93^ It has been reported that abnormal constitutional (i.e., meiotic recombination) and acquired (i.e., telomere attrition) have important effects on the generation of aneuploidy.^91^ On the one hand, according to the non-random loss hypothesis, the organism itself has a subset of autosomes with innate differences in acquired aneuploidy frequency, and the tendency of this chromosome to acquire aneuploidy increases with age.^91^ On the other hand, differences in telomere attrition rates due to oxidative stress can lead to chromosome loss, and the specific mechanism may be related to the localization of polymers in mitotic cells.^94,95^

#### Transcriptional alterations

Single-stranded RNAs (ssRNAs, including short ncRNAs (small interfering RNAs (siRNAs), microRNAs (miRNAs), circular RNAs (circRNAs), PIWI-interacting RNAs (piRNAs), endogenous siRNAs (endo-siRNAs), and long ncRNAs (lncRNAs)) mainly function as probes, antisense (AS) probes, miRNA analogs, and miRNA inhibitors and have great potential for gene therapy and molecular diagnosis.^96,97^ Double-stranded RNA (dsRNA) is produced by cells in the normal process of gene expression and is mainly derived from primary transcripts, which are either present in mature species or removed by RNA processing.^98^ Increasing evidence suggests that ssRNAs and dsRNA generated from the unstable genome are related to the aging process.^99,100^ For example, the levels of exosomal miRNAs secreted by hypothalamic brainstem/progenitor cells decrease during aging, while the aging rate is significantly slowed after treatment with exosomes secreted by healthy hypothalamic brainstem/progenitor cells.^101^ In addition, abundant circRNAs bearing miRNA response elements (MREs) may sponge miRNAs and hence lead to target mRNA repression.^102^ Recent profiling of genome-wide circRNAs has demonstrated that the expression of circRNAs is enhanced during aging in the brains of multiple organisms, but no other class of transcripts has been demonstrated to exhibit a correlation with senescence as strong as that of circRNAs.^103^ This may be because age-related elevations in global circRNA levels might originate from the high stability of circRNAs.^103^ Furthermore, the accumulation of dsRNA consisting of Alu sequences causes geographic atrophy, an advanced form of age-related macular degeneration caused by degeneration of the retinal pigment epithelium (RPE).^99^ In addition, due to changes in heterochromatin, neurodegeneration-associated proteins are tightly related to the derepression of repetitive element transcription.^100^ Saldi et al. reported that this derepression results in an elevation in the level of intracellular dsRNA, which activates innate immune responses and induces the neuroinflammation found in nearly all age-related neurodegenerative diseases.^100^

### Loss of proteostasis

Aging and various neurodegenerative diseases (e.g., AD and Huntington’s chorea) are mostly associated with impaired proteostasis.^104^ Proteostasis disruption triggers adaptive changes in the cell.^104^ To cope with this situation, cells have developed multiple mechanisms to reduce misfolding and remove misfolded proteins.^104^ One of these mechanisms is the development of chaperones, which bind to unfinished peptide chains. This prevents the peptides from folding prematurely and helps them fold into the correct shape.^104^ Chaperones also reduce the denaturation of proteins that occurs when cells experience heat shock; hence, these proteins are also called heat shock proteins.^105–107^

The endoplasmic reticulum (ER) initiates the unfolded protein response (UPR), which contributes to protein degradation and selective translation.^108^ Once the degradation and recycling systems downstream of the UPR (including the ubiquitin–proteasome system (UPS) and autophagy–lysosome system) become disordered, loss of proteostasis eventually occurs.^108^ Activating transcription factors (ATF3 and ATF4) that regulate UPR pathways play a key role in the aging process.^89,109^ In macrophages, inhibition of ATF3 leads to an elevated percentage of senescent macrophages in response to *Pseudomonas aeruginosa* PAO1 infection.^109^ ATF3 is also reported to remodel accessibility in senescence‐specific increased accessibility regions (IARs) in HUVEC senescence.^89^ Sun et al. reported that loss of ATF4 diminishes hematopoietic stem cell (HSC) function with an aging-like phenotype and impairs leukemogenesis by targeting HIF1α and p16^Ink4a^.^110^ During aging, proteasomal targets accumulate due to reduced ubiquitination and subsequent degradation.^107^ An elevation in the expression of the proteasomal target intermediate filament protein ifb-2 (IFB-2) has been shown to enhance the loss of intestinal integrity and bacterial colonization, while upregulation of epidermal growth factor receptor pathway substrate 8 (EPS8) hyperactivates AKT serine/threonine kinase 1 (RAC) in muscle cells and neurons.^107^ Hence, reducing the levels of age-dysregulated proteasomal targets can improve longevity. Koyuncu S et al. have reported that ubiquitin-coding gene expression is not downregulated in aged wild nematodes. This finding suggests that differences in ubiquitination levels are not due to differences in ubiquitin-protein expression but rather are due to loss of ubiquitination modifications.^107^ Insulin-like growth factor-1 (IGF-1) is another important anabolic growth factor that promotes protein synthesis via p70S6 kinase and p90 ribosomal S6 kinase and inhibits protein degradation mainly by suppressing proteasomal–lysosomal protein degradation.^111^ IGF-1 is a known driver of aging.^112^ In worms and flies, inhibition of the IGF-1 signaling pathways has been demonstrated to increase lifespan.^113^ In addition, in cultured cardiomyocytes, IGF-1 induces senescence, and a PI3K inhibitor abolishes this effect.^112^ However, a decrease in the expression of IGF-1 leads to declines in skeletal muscle quality and strength during aging.^114^ It has been suggested that IGF-1 contributes to skeletal muscle protein synthesis via the PI3K/Akt/mTOR and PI3K/Akt/GSK3β pathways, which then leads to UPS-mediated protein degradation.^115^ Thus, maintaining some level of IGF-1 is important for health, and extreme reductions in IGF-1 levels are detrimental for mammals.^116^ Small ubiquitin-related modifier (SUMO) is a newly discovered ubiquitin-like molecule.^117^ SUMO modification is similar to ubiquitination, but SUMO proteins do not mediate the degradation of target proteins. Instead, SUMO modification increases stability.^117^ Emerging evidence shows that alterations in global protein sumoylation and changes in the sumoylation pathway are extensively involved in the process of organismal aging.^118,119^ For instance, silencing of the sole SUMO gene (smo-1) results in a shortened lifespan, while overexpression of smo-1 extends the lifespan in *C*. *elegans*.^120^

In addition, ribosomes have been shown to play an important regulatory role in proteostasis.^121^ A recent study has suggested that as cells age, ribosomal translation “pauses” increase in frequency. This change leads to ribosome-associated quality control overload and *de novo* peptide aggregation, thus exacerbating protein aggregation.^121^

### Compromise of autophagy

Among the molecular changes associated with aging, alterations in autophagy have become recognized as important features of aging in different species.^25^ Autophagy is a highly conserved process that degrades cellular components, including defective organelles and misfolded protein aggregates, in lysosomes.^25^ It is mainly initiated by mTOR inhibition or adenosine monophosphate-activated protein kinase (AMPK) activation. There is increasing evidence that autophagic activity decreases with age in different tissues in different species.^25,122^ Lysosomal protein hydrolysis is reduced during aging, which impairs autophagy, exacerbates cellular damage, and promotes the development of age-related diseases.^123^ For example, in human neuronal cells, the expression of autophagy-related genes (e.g., ATG5, ATG-7, and BECN1) decreases with age.^124,125^ Conversely, increased autophagy is associated with delayed aging.^125^ For example, increased lifespan in *C*. *elegans* is associated with increased expression of the autophagy genes ATG-1, ATG-7, and ATG-18.^25^ These findings suggest that the abundance of autophagy-related proteins gradually decreases with age and that translocation to lysosomes is reduced, implying that impaired autophagy is a major feature of organismal aging.^25^

**Fig. 3:**
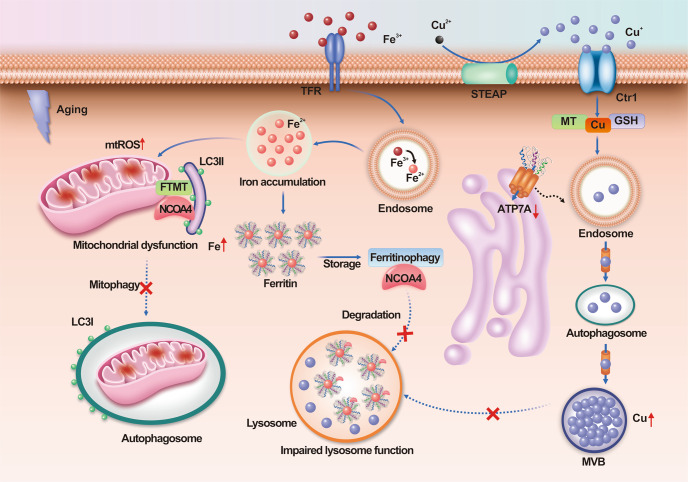
Iron and copper accumulate in senescent cells. In senescent cells, iron accumulation is due to defective autophagic degradation of ferritin by lysosomes. In addition, in aging cells, FTMT accumulates on the outer membranes of defective mitochondria and promotes mitophagy by specifically interacting with the autophagic cargo receptor NCOA4 coupled to the LC3-II double-membrane phagophore. Furthermore, in senescent cells, reductions in the levels of Atp7a (a copper exporter) block autophagic–lysosomal degradation of copper. Atp7a copper transporter copper-transporting ATPase 1, Ctr1 copper transporter 1, FTMT mitochondrial ferritin, LC3 I cytosolic form of LC3, LC3-II LC3-phosphatidylethanolamine conjugate, mtROS mitochondrial ROS, MVB multivesicular body, NCOA4 nuclear receptor coactivator 4, TFR transferrin receptor

Recently, researchers found much higher levels of transition metals (e.g., iron and copper) in senescent cells than in nonsenescent or immortalized cells (Fig. 3).^126,127^ Protein degradation pathways, such as autophagy and the UPS pathway, participate in the regulation of ferroptosis.^128^ For instance, iron accumulation is due to defective autophagic degradation of ferritin in lysosomes.^126^ In addition, mitochondrial ferritin (FTMT) accumulates on the outer membranes of defective mitochondria, which then promotes mitophagy, a specific form of autophagy that modulates the turnover of damaged and dysfunctional mitochondria, via specific interaction with the autophagic cargo receptor nuclear receptor coactivator 4 (NCOA4) coupled to the LC3-II double-membrane phagophore.^129^ In addition, enhanced levels of copper in senescent mouse embryonic fibroblasts (MEFs) and astrocytes have been reported to be accompanied by elevations in the levels of high-affinity copper transport protein 1 and reductions in the levels of copper-transporting ATPase 1 (Atp7a) (a copper exporter).^130,131^ Further research has shown that a lack of functional Atp7a blocks autophagic–lysosomal degradation of copper in senescent MEFs, thereby promoting aging-associated degenerative disease.^131^

### Mitochondrial dysfunction

With aging, mitochondria become highly susceptible to morphological changes. These changes result in reduced function due to oxygen radical damage, which eventually causes the aging of the organism.^132^

#### Reactive oxygen species (ROS)

Mitochondria are major sources of ROS.^133^ In each individual, there is a concentration threshold between beneficial and detrimental ROS, named the redox-stress signaling threshold (RST), below which redox stress is beneficial.^134^ In *C. elegans*, starvation (or heat stress or exercise) stimulation increases RST, and increasing RST improves Redox-stress response capacity (RRC) and health span, suggesting that increasing RST values through early stimulation can effectively delay aging.^134^ Moreover, in various tissues of mice, naked mole rates (NMR), and bats, the mild depolarization of mitochondria has been proven to inhibit the production of mitochondrial ROS (mROS).^135^ In different organs of aging mice (skeletal muscle, diaphragm, heart, spleen, and brain), the mild depolarization of mitochondria generally disappeared.^135^ In long-lived NMR and bats, however, the mild depolarization of mitochondria remained unchanged.^135^ This result shows that ROS-mediated protein damage caused by the disappearance of mild depolarization of mitochondria is one of the main causes of aging in short-lived mice, and mild depolarization of mitochondria is crucial to the mitochondrial antiaging system.^135^ ROS have been reported to be important for maintaining tissue-specific physiology through reversible modification of protein cysteine residues, and aging-induced dysregulation of ROS and redox signaling causes a decline in tissue physiological function.^136,137^ Xiao et al. performed an in-depth analysis of the cysteine oxidation networks, the Oximouse dataset, a dataset that quantifies the percentage of reversible modifications at approximately 171,000 cysteine sites in ten tissues of young and aged mice.^138^ They found that aging tissues did not show an overall increase in protein oxidation levels; instead, cysteine oxidation networks were radically remodeled in all aging tissues.^138^ Unlike the conventional notion advocated that ROS-driven protein modifications increase with age, Oximouse’s results found that different redox signaling networks are selectively altered in different tissues.^138^

#### Mitochondrial energy metabolism disorder

Aging-related mutations in mtDNA cause defects in mitochondrial oxidative phosphorylation (OXPHOS) functions.^139^ In mice, doxycycline-induced mutations reduce mtDNA levels, alter mitochondrial gene expression, and destabilize the complexes involved in OXPHOS in mitochondria, in turn promoting skin aging and hair loss.^140^ Age-dependent decreases in NAD+ levels have been identified in several pathologies.^141^ In preclinical models, supplementation with NAD(+) extends health span and improves several conditions, such as premature aging diseases (Cockayne syndrome, CS) and neurodegenerative diseases.^142,143^ Mechanistically, replenishing intracellular NAD+ promotes DNA repair and enhances mitochondrial quality via mitophagy.^144^ In the mitochondria, NADH is oxidized into NAD+ by the electron transport chain (ETC), and this process is coupled to ATP synthesis.^145^ However, mitochondrial dysfunction may lead to ATP depletion and cellular senescence by decreasing the NAD+/NADH ratio in the cytoplasm and promoting ROS production, a process known as mitochondrial dysfunction-associated senescence (MiDAS).^146^ Even though the NAD precursors has been viewed as an antiaging drug, the paradoxical results are reported. It is suggested that the supplement of NAD precursors alone does not improve the insulin sensitivity, mitochondrial respiration, energy metabolism, ectopic lipid accumulation, and plasma inflammatory markers of healthy overweight or obese individuals, which may be due to the insufficient supplement time and dose of NAD, resulting in the limited improvement of metabolism in overweight people.^147–149^

#### Mitochondrial quality control imbalance

Mitochondrial quality control is an important factor in the maintenance of mitochondrial function that mainly includes the biogenesis of mitochondria and the biodegradation of damaged mitochondria.^150^ Mitophagy, a selective type of autophagy, specifically degrades damaged or redundant mitochondria within the cell.^151^ Changes in the expression of mitophagy-related proteins can affect the degradation of damaged mitochondria and are closely related to cellular senescence.^151^ The mitochondrial-derived vesicle pathway is a newly identified mitochondrial quality control pathway that helps maintain stable mitochondrial function during the early stages of cellular stress and plays an important role in mitochondrial oxidative stress.^152,153^

The above results suggest that the dynamic balance between mitochondrial biogenesis and degradation is essential for mitochondrial quality control. A reduction in the level of biogenesis and/or degradation can cause cellular senescence; thus, mitochondrial quality control is a target of interest for antiaging actions.

### Cellular senescence

Cellular senescence can be divided into two categories: replicative senescence and stress-induced premature senescence.^154^ Replicative senescence refers to the premature senescence that occurs after a limited number of divisions and a gradual shortening of telomeres at the ends of chromosomes. This results in cell proliferation stagnation and loss of differentiation ability.^154^ Stress-induced premature senescence refers to the premature senescence that occurs in response to pathological stimuli, such as DNA damage and oxidative stress.^154^ These two types of senescence share many regulatory molecules, and they both cause cell cycle arrest mainly through the p53/p21 and p16Ink4a/retinoblastoma protein signaling pathways.^155,156^ The number of senescent cells increases with age, obesity, and diabetes, and clearing senescent cells can alleviate many aging-related diseases and prolong the lifespan in mice.^155,157^ Senescent cells induce the formation of a complex, multicomponent SASP by secreting a range of cytokines, inflammatory factors, and adhesion factors.^158^ In the local microenvironment, the SASP alters the biological behavior of adjacent cells through autocrine and paracrine signaling.^158^ For instance, a systemic environment that shapes aging-related diseases has been reported to exist, as the infusion of young cerebrospinal fluid (CSF) improves the memory function of aged brains.^159^ This improvement is mainly mediated by serum response factor (SRF)-mediated oligodendrocyte progenitor cell (OPC) proliferation after exposure to young CSF.

Elderly individuals often present with chronic low-grade inflammation, which is collectively referred to as immune aging.^160^ In the process of aging, the numbers of monocytes/macrophages, dendritic cells, nd natural killer (NK) cells increase, possibly because of an increase in the number of aging cells, and results in increases in the numbers of macrophages and NK cells, thereby eliminating aging cells.^108^ Immune cells may further induce the production of more proinflammatory cytokines and aggravate the progression of aging-related diseases.^108^ In contrast, T-cell senescence may be one of the main features of immune senescence. Premature T-cell failure may accelerate aging in multiple organs and systems, with thymic degeneration, mitochondrial dysfunction, genetic and epigenetic alterations, and imbalance in protein homeostasis being the four main hallmarks of T-cell senescence.^161^ Desdín-Micó G et al. reported that in mice with mitochondrial transcription factor A (TFAM) deletion, T cells with mitochondrial dysfunction induce a variety of aging-related phenotypes, such as metabolic disorders, cognitive impairment, and cardiovascular diseases, which ultimately lead to the premature death of mice.^162^

### Stem cell exhaustion

Stem cells, with their potential for self-renewal and multidirectional differentiation, are core components of regenerative medicine. They have been used in the treatment of a variety of diseases, including hematopoietic, central nervous system, and immune system disorders.^163^ Autophagy is necessary to maintain the stemness and differentiation capacity of stem cells, but autophagy is impaired during stem cell aging.^164^ With aging, autophagy of bone marrow mesenchymal stem cells (MSCs) and osteoblasts decreases. Activation of autophagy can alleviate the aging of bone marrow MSCs and restore osteogenic differentiation and proliferation in senescent bone marrow MSCs.^165^

Theodore T. Ho et al. found that hematopoietic stem cells (HSCs) that are unable to undergo cellular autophagy have a buildup of mitochondria and are in a constant state of metabolic activation, which accelerates the differentiation of myeloid cells through abnormal DNA modifications, ultimately affecting the ability of HSCs to self-enhance.^166^ Furthermore, recent studies have shown that the expansion, depletion or maintenance of the stem cell pool are regulated through symmetric and asymmetric division events.^167–169^ Failure to properly control cell division patterns can lead to premature depletion of the stem cell pool or to abnormal growth and differentiation disorders that accelerate cellular senescence.^168^ Cell polarity proteins are potential key regulators of asymmetric cell division, and a reduction in or loss of asymmetric cell division may be associated with diseases common to the aging process.^169^ In mice, senescence damages CD8^+^ T asymmetric cell division and affects long-term T-cell survival and function, but this phenotype can be reversed by inhibition of mTOR.^170^ In addition, cell size is also reported to be a determinant of stem cell potential during aging. Murine and human HSCs enlarge during the aging process, which may result in reduced proliferation and altered metabolism and may ultimately reduce stem cell function.^171^

### Altered intercellular communication

Intercellular communication is typically characterized by the release of soluble factors and affects the function of neighboring cells.^172^ In the tissue microenvironment, the SASP has a range of negative effects on neighboring cells, the surrounding extracellular matrix and other structural components, including chronic inflammation and passive senescence of healthy cells.^173^ Extracellular vesicles (EVs) are lipid membrane vesicles that can be released by all cells and are well-established mediators of intercellular communication.^174^ Specific mesenchymal cells transfer specific processed tRNAs directly to granulocyte-/monocyte-lineage hematopoietic progenitors via EVs to promote protein translation, cell proliferation and eventual differentiation in granulocyte–macrophage progenitors.^175^ This unique form of stress-regulated communication may alter the physiological state of the organism in response to challenges, including infection.^175^ In addition, EVs exchange protein and lipid signals between endothelial cells and adipocytes, transmitting information regarding changes in the nutritional status of blood in adipose tissue.^176^

Senescent cells also communicate with each other and with neighboring cells in a cell-to-cell or proximal-secretory manner.^177^ Juxtacrine signaling is a form of intercellular communication that relies on the binding of receptors to ligands.^177^ For example, a juxtacrine NOTCH-JAG1 pathway induces senescence in oncogene-induced senescent cells.^178^ Cell‒cell fusion is a form of intercellular communication that induces senescence not only in primary cells but also in other cells.^179^ Bone marrow cell-derived TNF-α promotes muscle aging by affecting the fusion of muscle cells with aging muscle fibers.^180^ In addition to cell fusion, cytoplasmic bridges enable the intercellular exchange of biological materials, including RNA, proteins, and even organelles such as mitochondria and lysosomes.^181^ It was recently observed that mitochondria in senescent cells can be transferred to neighboring cells via a large number of membrane-bound intercellular bridges or tunneling nanotubes, a process that is largely dependent on signals from the mTOR pathway.^182^ Ma et al. suggested that CR improves the aging-disturbed immune ecosystem by reversing abnormal cell‒cell communication patterns, such as excessive proinflammatory ligand‒receptor interplay.^183^ Recently, bulk RNA sequencing of 17 organs and plasma proteomics at 10 ages across the lifespan of *Mus musculus* demonstrated how gene expression shifts in distinct tissues are tightly related to the corresponding protein levels in plasma, promoting the aging of the systemic circulation, indicating the existence of a similar yet asynchronous inter- and intra-organ progression of aging.^184^

### Deregulated nutrient sensing

The somatotrophic axis is a neuroendocrine axis consisting of relevant hormones and receptors on the hypothalamus–pituitary–target organs that plays an important role in nutrient sensing and cellular energy perception.^185^ Nutrient perception refers to the ability of cells to recognize and respond to energy substrates, such as glucose, fatty acids, and ketones.^185^ Three key nutrient-sensing pathways are the insulin/IGF-1 signaling pathway, the mTOR pathway, and the AMPK pathway.^24^ In mammals, insulin/IGF-1 signaling is an important coordinator of nutrient availability with energy homeostasis and metabolic processes, which is activated by insulin-like peptide (ILP) ligands in response to nutrient availability.^116^ Insulin/IGF-1 signaling can initiate signal transduction via the PI3K/Akt pathway, which then phosphorylates many targets, including tuberous sclerosis complex (TSC) 1/TSC2, thereby regulating the activity of mTOR complex (mTORC) 1.^116^ Nutrients are key mTORC1 activators since they alone are enough to activate TORC1 in unicellular organisms, subsequently promoting anabolic processes including protein, lipid, and nucleotide synthesis and inhibiting catabolic processes such as autophagy.^186^ Mammalian AMPK is initiated by a falling cellular energy status and is activated by elevated AMP/ATP and ADP/ATP ratios.^187^ AMPK and mTOR signaling are interlinked and sense opposing nutrient states, regulate opposite metabolic processes, and regulate cell growth.^188^ Generally, AMPK turns off mTORC1 signaling when the energy status of cells is compromised.^188^ Dysregulation of insulin/IGF-1, mTOR and AMPK signaling is tightly related to human aging and age-related diseases due to nutrient insufficiency.^189,190^

The human microbiota contains multiple symbiotic microorganisms and participates in nutrient sensing.^25^ Thus far, bidirectional communication between the gut microbiota and the brain has been extensively found to occur through immune, circulatory and neural pathways in the so-called gut–brain axis (GBA).^25^ Disturbance along the GBA contributes to aging-related diseases.^191^ For instance, alterations in the gut microbiota composition enhance gut barrier permeability and systemic inflammation due to immune disorder, which then weakens the blood‒brain barrier (BBB) and induces neuroinflammation and ultimately neurodegeneration.^191^ Studies have found that the centenarian population has a unique intestinal flora composition from those of other populations, with certain intestinal flora and metabolites that produce unique secondary bile acids through new biosynthetic pathways.^192,193^ The gut microbiota plays a key role in immunity and metabolism due to its close association with other organs and tissues in the body.^194^ Aging-related alterations in the gut microbiota promote the development of systemic inflammation, which can have profound effects on disease, either directly or indirectly.^194,195^ For instance, microglia are the brain’s resident immune cells and regulate the survival of neurons and neuronal progenitor cells by secreting growth factors.^196^ However, the highly reactive and imbalanced state of microglia during aging causes cognitive dysfunction, including changes in brain plasticity and neurodegeneration.^196^ In germ-free (GF) mice, the microglial function can be improved by restoring key gut microbiota metabolites, such as short-chain fatty acids (SCFAs).^197^

## Summary

Aging is the result of a combination of physical, environmental, and social factors, so elaborating the molecular mechanisms that trigger aging is a daunting task. Human lifespan is closely related to the reduction of tissue and organ repair and regenerative potential. Specifically, at the molecular, cellular and systematic levels, genetic, epigenetic, and environmental regulatory factors cause a reduction in the physiological reserve of the organism in response to stress through complex molecular mechanisms that work together to promote aging. Molecular mechanisms (e.g., telomere shortening, accumulation of DNA damage, metabolic alterations, and excessive ROS production) link various factors closely to the rate of aging. Overall, these mechanisms stunt cell proliferation, alter metabolism and gene expression patterns and induce high levels of ROS production, maintaining the cellular senescent phenotype. Although the number of early senescent cells is not large, they can limit the regenerative capacity of tissue stem cells and induce the accumulation of cellular damage thereby promoting age-related diseases. Current developments in high-throughput genomics, proteomics, and metabolomics allow the characterization and quantification of thousands of epigenetic markers, transcripts, proteins and metabolites, and can reveal the overall changes that occur with age in complex organisms at the molecular level. Therefore, the integration of these molecular markers and related molecular mechanisms into a comprehensive assessment of biological age to counteract age-related functional decline and morbidity is increasingly becoming a hot issue of interest for scientists.

## Pathogenic and regulatory mechanisms of aging-related diseases

Aging is the most important risk factor for aging-related diseases. Therefore, the increasing age of the world population is accompanied by increases in the occurrence of various aging-related diseases. These diseases include neurodegenerative diseases, cardiovascular diseases, metabolic diseases, etc., all of which cause patients to lose normal life abilities, cause disabilities or even cause death. These diseases place a great burden on the social economy and the public health system. Next, we will focus on the regulatory mechanisms of aging and the pathogeneses of aging-related diseases.

### Alzheimer’s disease (AD)

AD is a progressive neurological disorder that causes problems with memory, thinking and behavior in elderly individuals. AD commonly occurs in individuals 60 years of age and older.^198^ AD is caused by progressive loss of neurons in the cerebral cortex and hippocampus, abnormal deposition of amyloid β-protein (Aβ) and the formation of senile plaques. Additionally, the hyperphosphorylation of tau proteins leads to the formation of senile plaques, which lead to impaired memory and reduced cognitive function.^198^

DNA mutations and defects in DNA repair mechanisms are important causes of AD. When DNA damage exceeds the repair capacity, mistranslation by DNA polymerase can lead to the development of neurodegenerative diseases.^199,200^ Due to aging and a reduced DNA repair capacity, DNA damage increases and accumulates in neurons. This leads to enhanced cellular oxidative stress and increased inflammatory responses. These processes trigger aging-related neurodegeneration and promote neuron senescence and AD.^201,202^

Epigenetic modifications, such as DNA methylation, PARylation, ubiquitination, and acetylation, also play important regulatory roles in AD progression.^203^ The brain is composed primarily of neurons and oligodendrocytes. Neurons cannot proliferate and are sensitive to epigenetic modifications caused by aging.^203^ Aging has been shown to alter DNA methylation processes. This leads to DNA damage, which may be responsible for neurodegeneration. Phosphorylation or hyperphosphorylation of histone H3 and deacetylation of histone H4 can be detected in the hippocampi of early AD patients. These findings suggest that epigenetic factors play an important role in the occurrence of AD.^204^

An increase in misfolded proteins and aggregation of tau proteins are also involved in the development of AD. Aβ oligomerization may block synaptic plasticity and signal transduction.^205^ Aβ can also surround mitochondria and impair their function, leading to the release of ROS, overactivation of microglia and generation of proinflammatory factors.^205^ Another important change is that Aβ self-aggregates and accumulates on neuronal membranes, generates ROS, undergoes membrane lipid peroxidation, and generates 4-hydroxy-2-nonenal.^206^ This, in turn, impairs the functions of membrane ion kinetic ATPase and glucose and glutamate transporters and disrupts neuronal Ca^2+^ homeostasis. These effects result in neuronal hyperexcitability, susceptibility to excitotoxicity and metabolic exhaustion, ultimately resulting in Aβ neurotoxicity.^206,207^ However, some other studies found that this hypothesis may be controversial.^208^ Recent research has found that cellular damage with AD characteristics appears in neurons before amyloid fragment accumulation and amyloid plaque formation. Further research has revealed that these nerve cells have autophagy disorders and cannot effectively decompose Aβ, leading to the corresponding phenotype.^209^

Tau proteins may also aggregate and form neurofibrillary tangles.^210^ Neurofibrillary tangles gradually accumulate in the brain and are closely related to the prevalence of AD and the degree of disease.^211^ Furthermore, dominant tau mutations lead to increased tau aggregation, neuroinflammation and neurodegeneration. These pathological tau conformations can recruit native tau proteins, induce the formation of more abnormally folded tau proteins, and further promote pathological fibrillar aggregation.^206^

Decreased mitochondrial quality and activity are associated with normal aging, neuronal mitochondrial dysfunction and energy deficits during AD development and promote Aβ and tau pathology. New findings suggest that the autophagy/lysosomal pathway that removes damaged mitochondria (mitophagy) is also compromised in AD, leading to the accumulation of dysfunctional mitochondria. Research in animal and cellular models of AD and in patients with sporadic late-onset AD suggests that impaired mitophagy triggers Aβ and tau accumulation through increased oxidative damage and cellular energy deficit, leading to synaptic dysfunction and cognitive deficits. These changes in turn impair mitophagy. Neurons require high levels of ATP to perform their physiological functions, so mitochondrial dysfunction also contributes to the development of AD.^212^ Mitochondrial dysfunction promotes tau phosphorylation through the activation of AMPK and excessive mitochondrial fission, which in turn impairs ATP production.^213^ In addition, the PTEN-induced putative protein kinase 1 (PINK1)-Parkin pathway is important in mitophagy and in neuronal mitochondrial dynamics and function.^214^ Dysregulated PINK1 and Parkin functions may decrease mitochondrial function, increase Aβ aggregation in AD brain cells, and decrease mitophagy function.^215^ Thus, interventions that improve mitochondrial quality and function may prevent neurodegenerative processes in AD.

In addition, AD mainly manifests as a large group of SASPs caused by abnormal secretion of growth factors, cytokines, ROS and metalloproteinases. Astrocytes are the largest population of glial cells in the brain and are involved in various physiological functions of the central nervous system. Senescent astrocytes exhibit decreased normal physiological function and increased secretion of SASP factors that contribute to Aβ accumulation, tau hyperphosphorylation, neurofibrillary tangle deposition, and neurological deficits in AD. The disruption of astrocyte functions may lead to a chronic inflammatory response and central nervous system pathologies, including impaired synaptic plasticity, BBB dysfunction, glutamate excitotoxicity, and a decrease in the number and proliferation of neural stem cells, leading to the development of neurodegenerative diseases such as AD.^216,217^

### Parkinson’s disease (PD)

PD is a chronic and progressive neurodegenerative disease with movement disorder in elderly individuals. Due to striatal dopamine deficiency, PD presents with dyskinesias, including impaired range and speed of movement, limb stiffness, or resting tremors.^218^ Dopaminergic neurons in PD often exhibit Lewy bodies, in which there is massive accumulation of α-synuclein (α-syn) in the cytoplasm.^218^

In PD patients, α-syn aggregation is widely regarded as a major causative factor. α-Syn oligomers form large, insoluble, neurotoxic fibrils called Lewy bodies. α-Syn oligomers can spread from cell-to-cell throughout the brain, thereby aggravating the progression of PD.^219^ Some studies have suggested that another potential mechanism for the pathological spread of α-syn is the binding of misfolded prefibrils to lymphocyte activation gene 3 (LAG3), which initiates the endocytosis, delivery, and cytotoxicity of α-syn prefibrils.^220^

Many studies have found that older PD patients have more severe impairment of dopamine function and higher levels of α-syn and tau proteins in the cerebrospinal fluid than younger people. To date, ~20 genetic mutations have been associated with PD, including missense mutations in SNCA (α-syn), PARK7, and LRRK2 and missense mutations or loss-of-function mutations in PINK1, PRKN, PLOG, and GBA.^221,222^ Therefore, recent pharmacological developments have focused on restoring striatal dopamine levels through gene- and cell-based approaches, and α-syn aggregation and cellular transport have been identified as the therapeutic targets with the greatest potential.^223^

Neuroinflammation has been an important target of drug intervention in neurodegenerative diseases. Increased numbers of senescent cells in PD patients are associated with increased SA-β-gal and p16 activity and sporadic α-syn deposition, leading to increased production of the proinflammatory cytokine interleukin-6 (IL-6). In addition to glial cells, fibrillar α-syn increases IL-1β secretion by interacting with TLR2, which is associated with NLRP3 inflammasome activation.^224^

### Heart failure (HF)

Currently, over 64 million patients worldwide have HF,^225^ and HF is a growing area of interest. Studies have suggested that cardiac aging is a critical risk factor for impaired cardiac function and the progression of HF.^226^ Senescent cardiomyocytes play pivotal roles in conduction abnormalities, increased pacing frequency, mitochondrial dysfunction, increased oxidative stress, and metabolic dysfunction.^227^ Interestingly, senescent cardiomyocytes have been demonstrated to maintain cell-intrinsic senescence and induce neighboring healthy cell senescence via paracrine signaling. Thus, senescent cardiomyocytes can promote inflammation and dysfunction.^228^ They can also promote the activation of cardiac fibroblasts in a paracrine manner and induce cardiac fibrosis. The SASP of senescent cardiomyocytes promotes HF progression. Therapies targeting the SASP could therefore also be used to treat HF-related pathologies.

Research has indicated that oxidative stress plays a major role in the pathophysiology of cardiomyocyte senescence, hypertrophic remodeling and HF.^229^ Consistent with this notion, increased ROS levels lead to irreversible cardiomyocyte damage, senescence and death by contributing to DNA and protein oxidative damage, lipid peroxidation, mitochondrial dysfunction, and cytochrome c release. These processes are strongly associated with severe cardiac dysfunction and HF progression.^230^ Oxidative stress can also disrupt mitochondrial integrity and eventually trigger a vicious cycle of mitochondrial impairment and oxidative damage.^231^ In addition, several reports have suggested that oxidative stress in cardiomyocytes induces the premature senescence of cardiac stromal cells, increases the recruitment of CCR^2+^ monocytes, and eventually contributes to an excessive inflammatory response and cardiac dysfunction.^232^ Moreover, several studies have shown that aging-related increases in ROS levels contribute to mitochondrial dysfunction, metabolic imbalance and irreparable cardiomyocyte damage via accumulation of mtDNA damage and mutations, which could lead to HF.

The heart is a well-recognized organ with extremely active energy metabolism. Due to the inefficiency of the heart in storing ATP, cardiomyocytes must continuously generate ATP. Recent studies have suggested that cardiomyocyte senescence could have major adverse effects on multiple aspects of energy metabolism, reduce the heart rate and result in HF. However, miR-195 could regulate the metabolism of the failing myocardium by altering the Sirtuin 3 (Sirt3) expression and the mitochondrial protein acetylation.^233^ Interestingly, activation of p53 signaling inhibits glucose transport and glycolysis via GLUT1 and GLUT4, which is the major glucose transporters in cells. Additionally, it causes the senescent phenotype in cardiomyocytes during aging.^234^

Epigenetic alterations have also been increasingly recognized as major contributors to the initiation and progression of cardiomyocyte senescence and HF. For example, recent studies have shown that overexpression of the histone demethylase KDM4D in cardiomyocytes through upregulation of genes involved in proliferation and the cell cycle can delay cell cycle exit and profoundly promote cardiomyocyte proliferation.^235^ Furthermore, interestingly, second-generation sequencing analysis has revealed that changes in the accumulation of m6A RNA methylation exceed changes in both mouse and human gene expression during the pathophysiological processes present in HF.^236^ Fibroblast growth factor 20 is a member of the fibroblast growth factor family and plays key roles in regulating cell autophagy, inflammation, senescence and apoptosis. For instance, fibroblast growth factor 20 reduces pathological cardiac hypertrophy by activating the signaling pathway of the deacetylase SIRT1, inducing deacetylation of FOXO1 and reducing oxidative stress.^237^

In addition to senescent cardiomyocytes, senescent nonmyocytes in the heart, such as endothelial cells, can also be observed in HF.^238^ Myocardial hypertrophy and interstitial fibrosis have been found to occur in aging-accelerated mice at 24 weeks of age. These conditions could lead to systolic and diastolic dysfunction and thereby drive HF. Moreover, the senescence-associated hallmarks of endothelial cells, including p53 acetylation and senescence-associated β-galactosidase (SA-β-gal) activity, are significantly upregulated.^239^ Accumulating evidence suggests that increased expression of p53 and p16, which are markers of aging, can induce cellular senescence and atrial fibrillation. In addition, a study has shown that in atrial appendages of patients undergoing cardiac procedures, increased levels of endothelial nitric oxide synthase can promote many cardiovascular phenotypes, including atrial fibrillation, during endothelial cell dysfunction.^240^

Similarly, multiple types of chemotherapeutic agents, including anthracyclines, can result in the senescence of many types of cells in the heart in the clinical management of cancer. This effect is achieved via the induction of severe DNA damage and cardiac mitochondrial dysfunction in cells, which ultimately leads to HF. For example, SIRT1 mRNA and protein levels are decreased, and the activation of AMPK is inhibited. This, in turn, enhances inflammatory stimulation in doxorubicin-induced senescent vascular smooth muscle cells (VSMCs).^241^ The accumulation of senescent cardiomyocytes promotes cardiac aging and the development of HF; however, autophagy-regulating protease 4a performs essential biological functions that facilitate mitochondrial function and subsequently inhibit doxorubicin-induced cardiomyocyte senescence.^242^

### Atherosclerosis

Vascular aging refers to aging-induced structural and functional changes that occur in the vasculature. Dysfunction of the vasculature contributes to aging-related diseases such as atherosclerosis, giant cell arteritis and AD, and is one of the leading causes of morbidity and mortality in elderly individuals.

Endothelial cells tightly regulate vasodilation by secreting vasoactive substances and growth factors. Senescent endothelial cells can be observed in atherosclerosis. The increased production of endothelin-1 and decreased production of nitric oxide in senescent endothelial cells lead to vascular inflammation and impaired vasodilation, compromise vascular endothelial integrity, and lead to vascular aging and atherosclerosis. Therefore, the accumulation of senescent endothelial cells can lead to vascular dysfunction, and vice versa.^243^

Oxidative stress is one of the main mechanisms driving atherosclerosis. Endothelial senescence can be triggered by oxidative stress or vascular inflammation. Nuclear factor E2-related factor 2 (Nrf2) is a key transcription factor that regulates hundreds of antioxidant genes and cytoprotective genes. Studies have shown that Nrf2 function is defective in atherosclerosis, hypertension and HF, and these conditions increase oxidative stress and accelerate aging.^244^ In addition, small extracellular vesicles derived from MSCs attenuate oxidative stress-induced endothelial cell senescence and stimulate angiogenesis through miR-146a/Src.^245^

Epigenetic changes, such as miRNA binding or histone acetylation, also contribute to endothelial cell senescence. An increasing amount of evidence suggests that miRNAs play important roles in the pathogenesis of vascular aging and atherosclerosis, and identification of aging-related miRNAs may provide opportunities for the treatment of cardiovascular disease. For example, in ApoE^−/−^ mice, miR-217 causes endothelial cell dysfunction and exacerbates atherosclerosis by downregulating endothelial nitric oxide synthase.^246^ In endothelial cells, miR-217 can also stimulate the senescent phenotype by downregulating SIRT1 expression.^247^ In the circulating blood of patients with atherosclerosis and hypertension, the numbers of some exosomes containing miRNAs that can induce endothelial dysfunction are significantly increased,^248^ which can increase the risk of atherosclerosis.^249^ Exosomes can accelerate the aging process by carrying miRNAs that promote cellular senescence to different tissues and organs, including those of the cardiovascular system. These aging-related exosomes may become biomarkers for some aging-related diseases and provide new targets for the treatment of these diseases in the future. In addition, the levels of SIRT1 and SIRT6, protein deacetylases that play key roles in regulating DNA damage repair, maintaining telomere length and metabolic homeostasis,^250^ are decreased in atherosclerosis.^251^ SIRT6 deficiency promotes endothelial cell senescence, leading to impaired vasodilation, vascular dysfunction and atherosclerosis.

Metabolic factors such as hyperuricemia and dysregulation of the renin–angiotensin system can also promote endothelial cell senescence. Prohibitin-1 is highly expressed in endothelial cells. This protein is mainly located in the inner mitochondrial membrane and plays important roles in mitochondrial biogenesis and the maintenance of mitochondrial function.^252^ Knockdown of prohibitin-1 leads to increased mitochondrial ROS production, which in turn leads to cellular senescence, cell migration, and impaired angiogenesis. This suggests that prohibitin-1 is involved in cardiovascular disease.^253^ Klotho is also a key molecule associated with aging. Klotho expression is reduced in mouse models of premature aging, which results in the development of atherosclerosis and greatly shortens lifespan. However, overexpression of Klotho prolongs lifespan.^254^

Similar to endothelial cell senescence, VSMC senescence also contributes to atherosclerosis.^255^ VSMCs coordinate with endothelial cells to control blood pressure, vascular tone, and blood flow.^256^ Thus, senescent VSMCs play an important role in the development of atherosclerosis. Studies have shown that both p16 and p21 expression and SA-β-gal activity are increased in plaque VSMCs.^257^ Telomere shortening, DNA damage, oxidative stress and epigenetic changes can induce VSMC senescence. For example, sustained DNA damage signaling promotes the secretion of pro-osteogenic cytokines, leading to VSMC senescence, mineralization, and subsequent vascular calcification.^258^ Plaque VSMCs also exhibit reduced SIRT6 expression, which can lead to hyperacetylation of H3K9 and H3K27. This, in turn, leads to telomeric DNA damage and VSMC senescence.^259^ Senescent VSMCs exhibit increased expression of inflammatory cytokines, such as C-C motif chemokine ligand 2 (CCL2), monocyte chemoattractant protein 1 (MCP1), macrophage inflammatory protein-1α/β and CCL3/4. These cytokines promote the recruitment of monocytes, macrophages and lymphocytes, thereby accelerating the risks of plaque growth and rupture.^260^ In addition, senescent VSMCs secrete IL-1, IL-6, and IL-8, and exhibit upregulated TLR4-mediated signaling, and downregulated expression of anti-inflammatory factors.^261^ The upregulation of IL-1α expression results in the activation of the SASP of neighboring cells and increased secretion of IL-6, suggesting that senescent VSMCs can induce local inflammation through paracrine effects.^262^ Accumulation of prelamin A in VSMCs leads to the clinical manifestations of Hutchinson-Gilford progeria syndrome. Patients with this syndrome suffer from severe atherosclerosis, which accelerates aging and causes premature death.^263^

Immune system functions decline with increasing age, and a decline in immune function is a major risk factor for some cardiovascular and neurodegenerative diseases. Blood vessels are special sites for the immune system. Blood vessels consist of endothelial cells, VSMCs, macrophages, dendritic cells, fibroblasts and pericytes. Thus, the senescence of multiple cells can affect vascular homeostasis. Increased proportions of Th17 and regulatory T cells are observed in atherosclerotic plaques.^264^ The imbalance between Th17 and regulatory T cells enhances autoimmunity and increases autoantibody production, which may cause further tissue damage, ultimately leading to immunosenescence and atherosclerosis.

Circulating endothelial progenitor cells (EPCs) are generated in the bone marrow and are important for maintaining endothelial integrity.^265^ Many studies have shown the presence of senescent EPCs in various cardiovascular diseases, such as atherosclerosis, hypertension, ischemic heart disease and HF, and these senescent stem cells are dysfunctional in repairing endothelial damage.^266^ EPCs also show dysfunction in diabetes due to abnormal glucose metabolism, which may be one of the reasons why diabetic patients are prone to complicated cardiovascular disease.^267^

### Type 2 diabetes mellitus (T2DM)

T2DM is a global health problem, especially for older adults. This disease is characterized by defective insulin secretion, hyperglycemia and hyperlipidemia. The incidence of T2DM is growing rapidly for people over the age of 65.^268^ In 2019, 111 million T2DM cases were reported for people in this age group.^269^ Aging and obesity are the predominant risk factors for T2DM, and an increased number of senescent β-cells is associated with the pathogenesis of T2DM.^157^

Pancreatic β-cells secrete insulin and maintain the balance of blood glucose and lipids. The senescence of β-cells leads to β-cell dysfunction, which impairs insulin secretion and the homeostasis of glucose and lipid metabolism.^270^ Telomere attrition is a hallmark of aging and is also a surrogate marker of senescent β-cells in T2DM.^271^ In pancreatic β-cells from T2DM patients, the telomere length is shortened, which may impair the proliferation and insulin secretion of β-cells.^272^ In mice with short telomeres, insulin secretion and glucose intolerance are impaired. The expression of p16(INK4a) and the number of senescent β-cells are increased in pancreatic islets.^273^ The loss of β- cell mass leads to fasting hyperglycemia and impairs mitochondrial membrane integrity.^273^ In a cancer-resistant mouse model, telomerase reverse transcriptase overexpression has been found to improve glucose tolerance.^274^

Elevated levels of plasma free fatty acids (FFAs) and glucose result in inflammatory factor and ROS accumulation, ER stress and mitochondrial dysfunction, which impair the proliferation of β-cells and adipose cells.^270,275,276^ ROS lead to the formation of advanced glycation end products and disturb proteostasis.^277^ Inflammation also leads to the dysfunction of β-cells and adipose cells. Circulating FFAs and glucose activate the TLR4-MyD 8 pathway and stimulate the production of proinflammatory factors and chemokines in β-cells, such as IL-1β, IL-6, IL-8, CCL2, and CXCL1.^278,279^ IL-1β attenuates insulin secretion and stimulates the activation of resident immune cells.^278^ The chemokines CCL2 and CXCL1 promote the recruitment of monocytes within the islets of T2DM patients and the differentiation of these cells into macrophages. In the islets of T2DM patients, macrophage infiltration is increased. These macrophages are prone to polarize toward the proinflammatory (M1) type.^280,281^

Insulin resistance is a major factor for the pathogenesis of T2DM, which accelerates β-cell senescence. In one T2DM animal model, senescent cells accumulated, proliferation was diminished, and the levels of senescence markers were increased in β-cells and adipose cells. These findings suggest that β-cell and adipose cell senescence might be associated with insufficient insulin secretion and the pathogenesis of T2DM.^282–284^ Senescent β-cells produce the SASP factors CCL4 and IL-6 and affect resident cells. Conditioned medium from β-gal-positive cells increases the expression of p16 in healthy β-cells.^283^ The β-cell SASP involves many proinflammatory factors that can lead to the senescence and dysfunction of neighboring cells through paracrine actions.^285^ Multiple SASP factors are transcriptionally upregulated in models of β-cell senescence, aging, insulin resistance, and T2DM.

Aside from β-cells, adipose tissue is the most important energy reservoir and endocrine organ, and it regulates the homeostasis of lipid and glucose metabolism. Many factors can lead to the senescence of adipose cells, such as telomere attrition, DNA damage, mitochondrial dysfunction, ROS, ER stress and inflammation. Adipose tissue contains a large number of immune cells, which are affected by different physiological environments, lifestyle factors, caloric intake and aging. Adipose cell senescence triggers inflammation and insulin resistance in other metabolic organs, which lead to T2DM.^286^ Moreover, changes in the composition of the gut microbiota are linked to the onset of T2DM through decreased glucose tolerance and insulin resistance.^287^

### Nonalcoholic fatty liver disease (NAFLD)

NAFLD has been linked to aging-related chronic liver disease, and the major characteristic of NAFLD is hepatocellular fat accumulation.^288^ NAFLD is classified into two categories according to liver pathology: nonalcoholic fatty liver (NAFL), which is also referred to as simple hepatic steatosis, and nonalcoholic steatohepatitis (NASH). NASH is associated with inflammation and fibrosis and can further progress to advanced cirrhosis and even hepatocellular carcinoma.^289^ Hepatocellular fat deposition results from an imbalance caused by enhanced de novo lipogenesis (DNL) and lipid absorption and decreased fatty acid oxidation and lipid secretion. NAFL progresses to NASH in approximately 10–25% of patients. Although the pathogenesis of NASH is not fully understood, lipotoxicity, oxidative stress, apoptosis, and inflammation have been suggested to promote the progression from NAFL to NASH.^288,289^

Genetic and epigenetic changes affect the pathogenesis of NAFLD. Genomic studies have reported that multiple single-nucleotide polymorphism (SNPs) are independently associated with the development and progression of NAFLD. In a cohort of individuals from the UK and Finland, the rs762623 variant within *CDKN1A* was found to be significantly associated with the progression of NAFLD.^290^ Additionally, multiple genome-wide association studies (GWASs) have verified that the rs738409 variant in the patatin-like phospholipase domain-containing protein 3 (*PNPLA3*) gene is associated with NAFLD progression.^291^ Epigenetic DNA methylation has also been verified to be associated with NAFLD progression. Loomba et al. found that a set of 152 differentially methylated CpG islands in the peripheral blood-DNA of NASH patients correlate with the severity of hepatic fibrosis and that this DNA methylation signature is associated with the age-related acceleration of NASH in patients.^74^ However, demethylation of histone H3K9 on the promoter of PPARγ2 may induce hepatic steatosis through upregulation of hepatic PPARγ2 expression.^292^

Impairment of mitochondrial function is a major factor that contributes to NAFLD.^293,294^ Hepatocytes are rich in mitochondria, which are responsible for energy metabolism. Mitochondrial dysfunction has been linked to a reduction in fatty acid β-oxidation (FAO) that is due to decreased carnitine palmitoyl transferase-1 (CPT-1) activity and decreased fatty acid clearance, resulting in the pathogenesis of NAFL.^295^ Moreover, impaired hepatic FAO is related to the progression of NAFLD in patients with obesity. During NAFLD, the impairment of FAO inhibits the activity of PPARα, resulting in hepatic lipid accumulation and inflammation.^296^ Liver mitochondria from aged mice produce more ROS and have a greater extent of dysfunction than those from young mice. The increased ROS levels may cause further FAO damage, which aggregates hepatic steatosis.^289^ Mitochondrial ROS production is linked to a reduced mitochondrial metabolic rate and decreased ETC activity.^297^ Impaired ETC activity may decrease ATP production and mitochondrial NAD^+^ levels, leading to p53-dependent cellular senescence.^298^ NAD^+^ plays a crucial role in the development of age-related NAFLD. Experiments in aged mice have verified that supplementation with the NAD^+^ precursor nicotinamide riboside (NR) alleviate hepatic lipid accumulation, improve liver function, and ameliorate mitochondrial dysfunction and fibrosis. These experiments have also demonstrated that these effects are SIRT2- and irisin-dependent.^299–301^

Chronic ER stress and UPR signaling play a major role in the aging process and are involved in NAFLD.^302^ The ER is the major organelle for lipid synthesis in hepatocytes, and a high concentration of intracellular lipids can activate ER stress by disrupting Ca^2+^ homeostasis. The accumulation of free cholesterol or phosphatidylcholine may affect sarcoplasmic reticulum/ER Ca^2+^-ATPase (SERCA) activity by changing the free cholesterol (FC)/phospholipid ratio of the ER membrane. This leads to decreased intra-ER Ca^2+^ concentrations and induces ER stress.^302^ Lipids can also directly activate ER stress via UPR sensors, especially IRE1ɑ and PERK. The transmembrane domains of IRE1ɑ and PERK may recognize the change in the unsaturated/saturated acyl chain ratio of the ER membrane.^303^ Upon ER stress, activated IRE1ɑ and PERK can stimulate proinflammatory and proapoptotic pathways. These pathways induce inflammatory reactions and hepatocyte apoptosis and promote NASH progression.^304^ Activated hepatic IRE1ɑ can increase the release of ceramide-rich EVs via XBP1-induced transcription of serine palmitoyl transferase, leading to the recruitment of monocyte-derived macrophages to the liver and to NASH in mice.^305^ In addition, cysteine-rich with EGF-like domains 2 (Creld2), which is a target of the UPR sensor ATF6, promotes tolerance to ER stress by augmenting protein folding. One study has verified that Creld2 deficiency results in dysregulation of the UPR and causes NASH during ER stress conditions in male mice.^306^

Autophagic function declines with increasing age. Lipophagy is a process of macroautophagy in which lipid droplets are selected for autophagic degradation.^307^ Lipophagy impairment has also been verified to occur in a cohort of NAFLD patients via histomorphological and molecular analyses.^308^ Chaperone-mediated autophagy (CMA) is a form of autophagy that regulates lipid metabolism by targeting the lipid droplet-associated protein perilipin 2/3 for degradation and then promotes lipophagy.^309^ Inhibition of key components in autophagy leads to lipid deposition in both mouse livers and hepatocytes. Both autophagic and CMA activity are impaired with aging, leading to lipid accumulation in various organs, including the liver.^310^ Furthermore, the accumulation of damaged mitochondria is a hallmark of NASH.^311^ Mitophagy, a form of autophagy that selectively targets abnormal mitochondria for lysosomal degradation, is important in hepatic homeostasis. With aging, impairment of hepatic autophagy in the fatty liver may fail to remove damaged mitochondria, therefore leading to activation of the mitochondrial death pathway and causing NASH via oxidative stress and apoptosis.^295^

In general, the liver has a remarkable capacity for regeneration and restoration. However, age-mediated changes impair the hepatic regenerative capacity. Senescent hepatocytes exhibit telomere attrition, which correlates with the progression of NAFLD.^298,312^ To date, the precise relations between cellular senescence and NAFLD have not been established, and cellular senescence may be both the cause and consequence of NAFLD. Senescence markers (p16, p21, and p53) have been identified in hepatocytes of NAFLD, and hepatocyte senescence correlates with the progression of NAFLD.^312,313^ Deletion of p16^Ink4a^-expressing senescent hepatocytes in INK-ATTAC mice significantly reduces hepatic lipid accumulation.^314,315^ Cyclin-dependent kinase-4 (CDK4) may be inhibited by p16 to maintain the senescent phenotype. However, the levels of CDK4 in NAFLD patients are increased in an age-dependent manner.^316^ In addition, senescent hepatocytes exhibit specific metabolic dysregulation and mitochondrial dysfunction, which have been linked to NAFL pathogenesis.^312^

Seventy percent of the blood that the liver receives is supplied from the intestine via the portal vein; thus, bacteria-derived molecules influence hepatic metabolism and the pathogenesis and progression of NAFLD through enterohepatic circulation.^317,318^ There is an association between gram-negative bacteria and the progression of NAFLD with fibrosis. Advanced NAFLD patients exhibit significant dysbiosis with increased abundances of Bacteroides, Escherichia, and Ruminococcus and a decreased abundance of Prevotella bacteria.^318^ Studies have shown that impairment of intestinal permeability leads to increased liver inflammation under high-fat diet administration. When microbial products reach the liver via the portal vein, toll-like receptors (TLRs) and nucleotide-binding oligomerization domain-like receptors (NLRs) in both parenchymal and nonparenchymal cells can be activated and trigger the innate immune system.^319^

Under insulin-resistant conditions, the amounts of nonesterified fatty acids (NEFAs) in the circulation that are derived from white adipose tissue lipolysis increase, resulting in fat overload in the liver.^291^ Moreover, hepatic fat overload causes mitochondrial dysfunction, worsening hepatic insulin resistance. Interestingly, under conditions of NAFLD-related hepatic insulin resistance, hepatic DNL remains activated in the absence of hepatic gluconeogenesis inhibition.^320^ Recently, after comparing obese NAFLD patients with obese-only patients, Horst discovered that the hepatic DNL increase is independent of hepatic insulin resistance and that the underlying molecular mechanism involves the activation of carbohydrate response element-binding protein (ChREBP). Activated ChREBP stimulates the glycolytic pathway, leading to an increase in the metabolic precursors for DNL.^321,322^ Additionally, insulin resistance may promote oxidative stress and inflammation, resulting in the progression of NAFLD. However, NAFLD further worsens hepatic and systemic insulin resistance.^323^

Human studies have revealed that there is a positive association between the number of macrophages and NAFLD severity. Hepatic macrophages include both resident macrophages (Kupffer cells (KCs)) and infiltrating monocyte-derived macrophages.^324,325^ Deletion of KCs prevents the progression of NAFLD, and inhibition of monocytes in the liver via blockade of C-C motif chemokine receptor 2 (CCR2) improves NASH.^326^ During NAFLD, activated KCs present M1-like proinflammatory activity and secrete cytokines. These cytokines induce inflammation and promote monocyte infiltration into the liver.^327^ Recent studies have found that monocyte-derived KCs appear in the liver during NASH. Further studies have shown that the self-renewal of resident KCs is impaired in NASH mice and that monocyte-derived KCs are generated to maintain KC numbers.^328,329^ Although the transcriptomic landscape between the two types of KCs is different, the lipotoxicity gene signature remains constant. This suggests that the cellular stress signature probably drives both the death of resident KCs and the generation of monocyte-derived KCs.^328–330^ In addition, monocyte-derived KCs localize to fibrotic areas in the liver and localize close to desmin^+^ hepatic stellate cells (HSCs) during NASH in mice, suggesting that they may participate in hepatic fibrosis.^331^

**Fig. 4:**
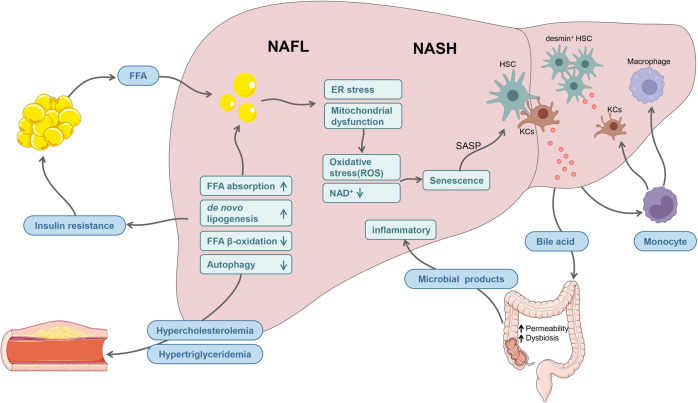
Crosstalk between aging and NAFLD in aging-related metabolic disease. Aging is related to impaired insulin sensitivity. Under insulin-resistant conditions, the amount of NEFAs in the circulation that are derived from white adipose tissue lipolysis increases, resulting in fat overload in the liver. Aging-related impairment of autophagy and mitochondrial dysfunction reduce hepatic lipid droplet breakdown and fatty acid β-oxidation, respectively. Moreover, under conditions of aging-related obesity, hepatic DNL increases due to ChREBP pathway activation. These disorders of lipid metabolism result in the pathogenesis of NAFL. Following hepatic lipid metabolic impairment and lipid accumulation, lipotoxicity and ER stress are induced, and mitochondrial function further worsens, leading to oxidative stress, hepatocyte apoptosis, hepatocyte senescence and inflammation and thus promoting the progression of NASH. Senescent hepatocytes secrete proinflammatory cytokines (IL-6, IL-8, TNF-α, and IL-1β) that stimulate resident KCs in the liver. Activated KCs present M1-like proinflammatory activity and secrete cytokines to induce monocyte infiltration into the liver and differentiation into macrophages. Furthermore, impaired resident KCs can induce monocyte differentiation into monocyte-derived KCs to maintain the KC pool in the liver. Both resident KCs and monocyte-derived KCs interact with HSCs and activate HSCs to produce collagen. In addition, dysbiosis of the gut microbiota impairs intestinal permeability; thus, bacteria-derived molecules enter the liver via the portal vein and in turn influence hepatic metabolism and the progression of NAFLD. However, hepatic metabolic impairment and inflammation further worsen insulin resistance and dysbiosis of the gut microbiota. Moreover, hepatic lipid metabolic disorder results in hypercholesterolemia and hypertriglyceridemia, leading to accelerated progression of atherosclerosis. (Fig. 4 includes modified templates from Servier Medical Art (http://www.servier.com), licensed under a Creative Commons Attribution 3.0 Unported License.)

Therefore, aging-related hepatic fat metabolic imbalance results in NAFL. Hepatic accumulation of lipids induces mitochondrial dysfunction and ER stress and further causes oxidative stress, hepatocytic senescence, inflammation, and fibrosis. These factors lead to the progression of NAFLD. Insulin resistance and dysbiosis of the gut microbiota accelerate the development and progression of NAFLD. Impaired hepatic lipid metabolism and inflammation further worsen insulin resistance and dysbiosis of the gut microbiota and promote the progression of atherosclerosis (Fig. 4).

### Osteoarthritis (OA)

OA is a chronic inflammation-related disease characterized by joint pain, cartilage loss, and joint inflammation.^332^ Cartilage on the joint surface provides a smooth platefor the painless movement of the joint. Cartilage loss is an important pathological feature of OA.^333^ More than 80% of elderly individuals over 65 years of age suffer from OA, which causes disabilities in elderly individuals and places a heavy burden on patients and society.^334^

Chondrocyte senescence is one of the major risk factors leading to OA. Chondrocytes maintain the stability of the joint synovium by synthesizing or degrading extracellular matrix components, such as type 2 collagen and proteoglycan polymers. With the development of OA, chondrocytes begin to degrade collagen and proteoglycans by secreting matrix metalloproteinase (MMP) 13 and ADAMTS-5, which ultimately leads to cartilage calcification.^335–338^ Senescent chondrocytes have been found in the cartilage tissue of joint specimens obtained during total joint replacement surgery.^339^ In OA patients, the number of senescent chondrocytes in articular cartilage tissue increases with age.^340–342^

Telomere attrition is associated with the pathogenesis of OA.^343^ The number of ultrashort telomeres is increased at the lesion site and is significantly correlated with the severity of OA at the lesion.^344^ Oxidative stress disturbs the balance between catabolism and anabolism in articular cartilage, which leads to matrix loss in joints and to OA pathogenesis.^345^ With aging, the accumulation of oxidative stress in chondrocytes reduces cell viability and sensitivity to growth factors.^346^ ROS can activate the MAPK and PI3K/Akt signaling pathways and upregulate p53 and p21 protein levels in chondrocytes. This leads to apoptosis and an inflammatory response.^347^

Mitochondrial dysfunction in chondrocytes is also related to the pathogenesis of OA. In the chondrocytes of OA patients, the numbers of mitochondria are decreased, and the integrity of the mitochondrial membrane is impaired.^348^ Damaged mitochondria promote ROS production and MMP13 expression, leading to loss of the cartilage matrix in OA patients.^349,350^

Inflammation promotes metabolic reprogramming of chondrocytes in which glycolysis is upregulated and OXPHOS is downregulated. This leads to cartilage degeneration and chondrocyte senescence.^351,352^ The expression of glycolysis-related genes, such as Glut-1, hexokinase II and PKM2, is increased in the chondrocytes of OA patients.^353–355^ The acidic microenvironment (pH = 6.6) in joints induced by accelerated glycolysis inhibits the synthetic activity of chondrocytes and leads to cartilage degeneration. Metabolic reprogramming also leads to mitochondrial dysfunction.^351^ AMPK and mTOR regulate energy metabolism and inflammation. Thus, they can maintain homeostasis and participate in the pathogenesis of OA.^348,356^ Inhibiting AMPK promotes the expression of the inflammatory factors TNF α and IL-1 β and the degradation of the cartilage matrix in chondrocytes.^357^ Cartilage-specific knockout of sirt1 accelerates the progression of OA in mice.^358^ Senescent chondrocytes induce senescence in other chondrocytes in cartilage by releasing SASP factors, such as IL-6, IL-1β, and MCP1. The SASP recruits macrophages to infiltrate the synovium and activate the inflammatory response, which leads to synovitis.^359^

### Osteoporosis (OP)

OP is an aging-related bone disease that is characterized by bone mass reduction and bone microstructure damage.^360^ Normal bone remodeling requires a balance between bone formation driven by osteoblasts and bone resorption driven by osteoclasts. With aging, the anabolic pathway (bone formation) is downregulated, and the absorption pathway (bone resorption) is upregulated. Aging-related bone loss is caused by a reduction in the number of osteoblasts.

Genomic instability is a hallmark of aging, which also leads to aging-related bone mass loss.^24^ DNA damage can further lead to the senescence of osteocytes and osteoprogenitors. An abnormal DNA repair system accelerates cell senescence. In humans, abnormal DNA repair systems lead to progeria, which is characterized by abnormal bones and low bone mass.^361^ Telomere reverse transcriptase–knockout (Terc^−/−^) mice are characterized by accelerated aging bone, reduced numbers of osteoblasts, increased numbers of osteoclasts and an inflammatory bone microenvironment.^362–365^ In OP, excessive ROS induce the apoptosis of osteoblasts and osteocytes and inhibit mineralization and osteogenesis. This causes an imbalance between bone remodeling and bone loss.^366–368^ Accumulation of senescent cells in bones is one cause of OP. The expression level of p16 is elevated in B cells, T cells, myoid cells, osteoprogenitors, osteoblasts and osteoclasts of 24-month-old mice.^369^ Senescent bone cells can impair resident cells by secreting SASP components. SASP factors in the supernatant of senescent cell culture can promote the survival of osteoclast progenitor cells and inhibit osteoblast differentiation.^370^

MSCs located in the bone marrow and spongy bone are responsible for maintaining the balance of bone resorption and formation.^371^ Bone marrow MSCs are located in the bone marrow and can differentiate into osteoblasts, adipocytes or chondrocytes. Depletion of bone marrow MSCs is one of the main causes of OP in the elderly population.^372^ Stenderup et al. found that MSCs derived from aging individuals exhibit a decreased maximal lifespan and an increased proportion number of SA beta-gal+ cells.^373^ Bone marrow MSC senescence can be caused by telomere shortening, genotoxic stresses/DNA damage, strong mitogenic signals, oxidative stress, and distortions in chromatin organization.^374–379^ In senescent bone marrow MSCs, the ability to differentiate into adipocytes is increased, and the ability to differentiate into osteoblasts is decreased.^370,380^

### Chronic obstructive pulmonary disease (COPD)

COPD is a lung disease characterized by the presence of chronic bronchitis or emphysema that leads to the development of airflow limitations. The incidence rate of COPD is high in elderly individuals, especially those above the age of 65 years.^381,382^

Age is one of the main risk factors for COPD. The structural and physiological characteristics between aged lungs and COPD lungs overlap to a considerable extent; such characteristics include increases in the size of alveoli and end-expiratory lung volume without destruction to the alveolar wall.^382,383^ Additionally, the clinical features of premature lung aging can predict the possibility of being diagnosed with COPD in later years.^383^ Senescence-accelerated mice (SAM) and Klotho gene-knockout mice show phenotypes of COPD,^384,385^ indicating that aging accelerates the process of COPD.

The senescence of functional cells and the exhaustion of progenitor cell groups in aged lungs lead to a decline in lung function that is closely related to the progression of COPD.^383^ Decreases in the number and frequency of ciliary body beats in ciliated cells in the trachea, bronchus and bronchioles decrease mucus clearance in the lungs.^386^ A reduction in the number of type I alveolar cells results in the obstruction of gas exchange in the alveoli.^387^ Decreases in the ability of goblet cells, tuft cells and club cells to remove pathogens and cell debris increase the risk of infection.^388^ The reduction in the repair and renewal of these functional cells after the injury is closely related to the exhaustion of lung progenitor cells. Basal cells are multipotent progenitors located in the conducting airway that can differentiate into club cells and further differentiate into ciliated cells or secretory cells (goblet cells, etc.). Research has shown that the numbers of basal and club cells decrease with age. Alveolar type 2 cells form the main progenitor cell group of the lung parenchyma and can differentiate into alveolar type 1 cells. Although the number of alveolar type 2 cells remains unchanged, the self-renewal and differentiation capacity of these cells decrease.^383^ In addition, the changes in immune cell function and phenotype caused by aging promote the susceptibility of elderly individuals to COPD. During aging, the concentration of alveolar macrophages (AMs) in the respiratory tract declines, and phagocytosis and the scavenging capacity are impaired. This triggers nonspecific inflammatory reactions that recruit neutrophils and dendritic cells to inflammatory sites. Insufficient clearance leads to an aggravated inflammatory response. Increases in the levels of proinflammatory cytokines, such as IL-6, are associated with increased COPD obstruction and increased risks of COPD-related complications.

Autophagy alterations contribute to COPD.^389^ Decreased autophagy in the lung tissue of COPD patients is related to the severity of emphysema. In the COPD mouse model, the activity of TFEB, the main transcriptional regulator of autophagy and lysosomal biogenesis, is inhibited.^390^ Transgenic mice with reduced proteasome activity (β5T) show an emphysema phenotype.^391^ Exposure to cigarette smoke may activate autophagy, resulting in ciliary dysfunction and the death of airway epithelial cells.^392^ Autophagy differs in different cell types and cellular environments, making it difficult to target autophagy therapeutically.^392^

Many studies have shown that mitochondrial dysfunction accelerates COPD. In the airways, the lungs and blood of COPD patients show mitochondrial morphological alterations, mitochondrial dysfunction, and increased ROS levels.^393^ The mitochondria-specific antioxidant mitoQ can reduce airway smooth muscle cell inflammation and the airway response.^394^ Mitochondrial autophagy is also deficient in COPD patients. The levels of PARK2 protein are decreased in COPD lungs and are positively correlated with lung function. PINK1- and PARK2-deficient mice show mitochondrial dysfunction and a COPD phenotype.^395,396^

Epigenetic alterations also play a role in the process of COPD. Abnormal DNA methylation has been detected in small airways^397^ and lymphocytes^398^ in COPD patients. In COPD patients, the expression levels of the deacetylases SIRT1 and HDAC2^399^ are decreased in macrophages and/or lung tissue. These reduced levels fail to control downstream transcription factors, such as FoxO3 and Nrf2, resulting in an abnormal immune response.^400,401^ Knockout of the SIRT1 gene in mouse airway epithelial cells induces a COPD phenotype.^402^ A variety of ncRNAs are involved in the pathogenesis of COPD.^403,404^

The mTOR signaling pathway is highly activated in multiple cells in COPD lungs, which enhances the susceptibility to pulmonary inflammation^405^ and emphysema.^406^ Metformin-activated AMPK pathway ameliorates the COPD phenotype in mice,^407^ indicating that nutritional signals also affect the process of COPD.

Increased genomic instability and telomere dysfunction are associated with COPD. In COPD patients, the levels of DNA damage markers in lung tissue and peripheral blood cells are increased,^408,409^ telomeres are shortened,^387,410^ and the expression of DNA repair-related proteins is decreased,^411^ all of which are related to disease severity.^412^ By regulating the expression of telomere protective protein 1 (TPP1), telomerase reverse transcriptase and telomerase (TERC), we can improve cell senescence^413^ and COPD phenotypes in mice.^414^ In addition, aging cells release incremental proinflammatory cytokines through SASP reactions, resulting in chronic lung inflammation.^415^

### Benign prostatic hyperplasia (BPH)

BPH is a common urogenital disease in middle-aged and elderly men. Previous studies have indicated that increasing age is an important risk factor for the development of BPH. At present, the pathogenesis of BPH is believed to include upregulation of androgen receptor expression, increased levels of inflammation-promoting growth factors, metabolic syndrome, endocrine and neurotransmitter changes caused by oxidative stress and epithelial–mesenchymal interactions, and lifestyle and dietary habits. All of these mechanisms can lead to the proliferation and apoptosis of epithelial cells and stromal cells in prostate tissue. These effects result in an increase in the number of cells and eventually lead to the development of BPH.^416^

Changes in androgen levels and tissue remodeling caused by aging are generally considered to be the major determinants of BPH. There is clinical evidence that taking a 5α-reductase inhibitor can reduce the concentration of dihydrotestosterone in prostate tissue, thereby preventing the further development of BPH. Compared with controls, androgen-depleted animals have lower bacterial counts and inflammation, reducing the risk of BPH development.^417,418^

Recent research also suggests that BPH may be an immune-inflammatory disease. Approximately 90% of prostate immune cells are T lymphocytes, which promote the release of cytokines and growth factors. These factors further lead to abnormal remodeling of the prostate structure characterized by tissue injury, chronic immune responses, and fibromuscular growth. Autoimmune responses associated with T cells may induce abnormal proliferation of epithelial and stromal cells involved in epithelial–mesenchymal transition (EMT). Both epithelial and stromal cells of the prostate can upregulate proinflammatory signals and trigger an inflammatory response following a bacterial challenge. As inflammation progresses, macrophages and MCP1, IL-8, IL-1, transforming growth factor-β2, and C-C motif chemokine 3 accumulate locally. This accumulation can increase the rate of cell growth, aggravate inflammation,^419^ and promote the development of BPH.^420,421^ In one study, compared with the patients in a nonstromal group, the BPH patients with inflammation in the stromal group had larger prostate volumes, a higher incidence of acute urinary retention, and lower maximum urine flow.

A key to the development of BPH in elderly individuals is metabolic syndrome, which is associated with low testosterone and hyperestrogenism. The characteristics of metabolic syndrome include T2DM, hypertension, obesity, high insulin levels, and low high-density lipoprotein-cholesterol levels. Metabolic syndrome components alone are risk factors for the development of BPH. IGF-1 has been shown to have strong mitogenic and antiapoptotic effects on prostate tissue. As the prostate volume increases, the patient’s serum insulin level increases, and IGFBP-3/PSA levels decrease. These effects suggest that early interventions that improve the insulin level may help control BPH.^422,423^

### Aging-related macular degeneration (AMD)

AMD is a degenerative disease of the macula that leads to severe visual loss in the elderly population.^424^ Clinically, early AMD is characterized by the deposition of lipoproteinaceous drusen on the Bruch membrane accompanied by pigmentary abnormalities in the RPE and progresses into two late forms: dry (atrophic) and wet (neovascular) AMD.^425^

In the retina, senescence of the RPE, neurons, microglia, and endothelial cells accelerates AMD. Senescent RPE cells destroy the external blood‒retinal barrier (BRB) between the retina and the choroid, resulting in drusen deposition and progressive macular damage.^426^ Senescent resident neurons contribute to photosensitive system disorders.^427^ Senescent retinal vascular endothelial cells decrease the oxygen supply capacity,^427,428^ and senescent microglia lead to persistent inflammation and loss of tissue homeostasis.^429^

Aging of the immune system decreases inflammatory regulation ability and immune clearance. Senescent phagocytes (such as macrophages and neutrophils) show reduced phagocytic activity and clearance and induce the production of atypical lipid species in the retina. A variety of immune cells, such as monocytes, neutrophils and T cells, invade the retina.^430,431^ In addition to affecting cellular functions, senescent cells lead to retinal degeneration by releasing SASP components. Retina-derived SASP induces BRB matrix degradation,^428^ destroys the tight junction proteins^432^ of the retinal barrier, recruits and activates immune cells to increase inflammation or releases angiogenic growth factors and VEGF to participate in angiogenesis.^433^

Senescent cells also show abnormal metabolic regulation. For example, senescent macrophages show abnormal ABCA1-mediated cholesterol metabolism, which reduces drusen clearance and promotes retinal aging.^432^ In addition to macrophages, peripheral blood mononuclear cells have similar roles in lipid metabolism regulation.^431,434,435^ In RPE cells, NAD^+^, mediated by the AMPK/mTOR pathway, leads to photoreceptor degeneration^436,437^ by affecting the metabolism of glycophospholipids, lipids and proteins in the retina.^438,439^

Autophagy and protein homeostasis affects AMD progression. The expression of autophagy-related proteins and autophagy flux are decreased in the retinas of aged rats^440^ and AMD patients.^441^ Nrf2/p62-mediated autophagy regulate the formation and accumulation of drusen in RPE cells.^442^ The accumulation of drusen further drives lysosomal damage^443^ and mitochondrial autophagy dysfunction,^444^ resulting in a vicious cycle of damage.^445^ In addition, the UPR^446^ and sumoylation^447^ regulate the aging process of the retina and RPE by regulating the degradation of proteins involved in the cell cycle,^447^ autophagy,^448^ and cytokine processes.

Mitochondrial dysfunction is also associated with AMD progression. The RPE in elderly individuals and AMD patients shows a decrease in the number of mitochondria and impaired activity. In addition, the shift from OXPHOS to glycolysis causes RPE dysfunction and subsequent photoreceptor death.^449^ The humanin peptide encoded by the mitochondrial gene humanin plays a potential protective role in RPE cells by reducing oxidative stress. Humanin enhances mitochondrial function and biogenesis via increases in mtDNA mass, mitochondrial number, and the protein expression level of the mitochondrial transcription factor mtTFA, which is a key protein involved in mitochondrial biogenesis.^450^ The traditional drugs lutein^451^ and tudca^452^ and new lipid mediators called elovanoids^453^ can protect photoreceptors by ameliorating oxidative stress.

In addition, mtDNA and nuclear DNA damage,^454,455^ telomere dysfunction,^412^ methylation changes[46], and RPE stem cell senescence^456^ contribute to AMD progression.

### Presbycusis

Presbycusis, also referred to as aging-related hearing loss (ARHL), is a progressive form of sensorineural hearing loss that occurs with aging and is a common condition in the elderly population.^457^

Accumulation of dysfunctional mitochondria might promote presbycusis progression.^457,458^ Decreased mitochondrial complex IV activity has been observed in the cochleae of elderly presbycusis patients^459^ and SAMP8 mice.^460^ Cdk5rap1 knockout in mice induces premature hearing loss by inducing mitochondrial dysfunction and mitochondrial tRNA modification dysregulation.^458^ In addition, lactate dehydrogenase B–knockout mice show hearing loss at high frequencies that is due to the regulation of mitochondrial membrane potential and ATP levels.^461^ Mitochondrial biogenesis is decreased in the spiral ganglion neurons (SGNs) of aged SAMP8 mice and increased in senescence-accelerated mouse resistant 1 strain mice.^460^ Mitochondrial-mediated apoptosis is also involved in the pathogenesis of presbycusis. The expression of Bax in the cochleae of SAMP8 mice is increased,^460^ and deletion of Bak prevents the aging-related loss of SGNs and hair cells in C57BL/6 J mice and the occurrence of presbycusis.^462^ In addition, oxidative stress damages hair cells and destroys the structure of the cochlea.^457,463^ Some studies have found significant associations between presbycusis and polymorphisms of antioxidant-related enzymes in the cochlea, including CYP1A1, UCP21, and MSR.^464^ SOD1^465^- and Gpx1^460^-knockout mice demonstrate accelerated aging-related cochlear hair cell loss and an increased susceptibility to noise-induced hearing loss.

Aging destroys protein homeostasis in the inner ear and leads to alterations in ion and water homeostasis that result in ARHL-specific dysfunction^466^ Autophagy alterations contribute to AMD. During the senescence of SGNs in SAMP8 mice, lc3-II is upregulated with lipofuscin accumulation.^460^ Mitophagy is essential for cell survival and cochlear functions. Recently, BCL-2 interacting protein 3-like (Bnip3) and NIX knockout in mice was shown to promote presbycusis by downregulating mitophagy.^467^ In addition, miR-34a promotes presbycusis in mice by inducing mitophagy impairment.^468^

The role of inflammation in ARHL has attracted much attention.^469^ In humans, there is a significant correlation between a reduction in the hearing threshold and the levels of four systemic markers of inflammation (leukocyte count, neutrophil count, IL-6, and C-reactive protein).^470,471^ There is also an association between systemic inflammation and ARHL,^472,473^ and the levels of IL-1β and TNF-α^460^ and the number and morphology of macrophages in the cochlea showed aging-related changes.^474^ Transcriptome data from young and old C57BL/6 mice show that the expression of genes related to the immune response and inflammatory pathways is increased in old cochleae.^475^

Increased genomic instability is associated with the onset of presbycusis. Mice expressing error-prone mtDNA polymerase gamma (PolgD257A) or with POLGD knockout show defective mtDNA replication fidelity and premature aging, which leads to early-onset ARHL.^476^ Increased levels of mtDNA oxidative damage markers have been observed in the cytoplasm of cells in the cochleae of SAMP8 mice.^477^ In addition, ROS-induced DNA damage drives the aging of cochlear cells in SAMP8 mice and helps to accelerate presbycusis.^478^ mtDNA deletion and mutation are increased in the cochleae of ARHL patients,^479,480^ and deletion of mtDNA4977 is closely related to the severity of presbycusis.^481^

Epigenetic factors are also involved in the process of presbycusis. Altered methylation modifications of connexin,^482^ amino acid transporter^483^ and signaling pathway-related proteins^484^ contribute to the risks of presbycusis in different populations.

### Cancer

The link between aging and cancer is complex. Although there is clear evidence that cells entering a senescent state can act as a barrier to tumorigenesis, some studies have demonstrated that, in certain conditions, persistent senescent cells can acquire pro-tumorigenic properties.

Senescent cells can initiate both intrinsic and extrinsic mechanisms to inhibit tumorigenesis. Induction of stable growth arrest forms a natural barrier to tumorigenesis which works as a typical intrinsic antitumor manner. In Oncogene-induced senescence, aberrant activation of proto-oncogenes such as RAS fuels unscheduled DNA replication, causing malignant cellular growth. In turn, cells trigger firm proliferative arrest and senescence by initiating key cell cycle arrest genes such as TP53, p16INK4a, and p21, which counteract malignant growth.^485^ In PTEN loss-induced cellular senescence, cells activate mTOR-p53-mediated signaling to block the cell cycle, and induce a cellular senescence phenotype.^486^ In addition, cells undergoing senescence enhance tumor suppression by cell-extrinsic manner. Senescent cells promote senescence or death of neighboring cells through direct cell-cell interactions or inflammatory SASP factors release, causing increased production of ROS and a sustained DNA damage response, thereby also limiting the proliferation of neighboring precancerous or cancerous cells.^485^ In addition, SASP factors containing immune surveillance enhancers such as CCL2, IL-15, CXCL1, etc. that drive macrophages, lymphocytes and NK cells to recruit to tumor sites, which activate immune surveillance. Chromatin reader bromodomain-containing protein 4,^487^ P53-P21 axis, and NFκB signaling pathway^488^ regulate immune surveillance by regulating the expression and type of SASP.

However, studies in the past decade also proved established that the persistent senescent cells induce pro-tumorigenic effects by producing a proinflammatory and immunosuppressive microenvironment.^485^ Due to the diversity and highly dynamic nature of SASP factors, different SASP factors may have opposite functions on each other, or some SASP factors may have contradictory multiple functions on themselves, which results in SASP factors having both tumor-suppressive and tumor-promoting effects. SASP factors secreted by senescent cells include pro-proliferation factors such as IL-6 and IL-8,^489^ tissue angiogenic remodeling factors such as VEGF and CXCL5,^490,491^ and pro-cancer metastasis and invasion factors such as MMPs and GDF-15.^492,493^ These factors drive tumorigenesis by creating a chronic inflammatory microenvironment that supports cancer growth, disrupting the extracellular matrix and promoting tissue vascular remodeling effects. SASP factors-driven immunosuppression is also responsible for the pro-tumorigenic effects of senescent cells. For example, IL-6 causes the recruitment of myeloid-derived suppressor cells to the tumor microenvironment,^494^ and myeloid-derived suppressor cells not only blocks IL-1-mediated cellular senescence, but also reduces immune surveillance through the suppression of antitumor cells such as CD8^+^ T cells and NK cells.^495^ Hepatocytes with NrasG12V-induced senescence attract a subpopulation of CCR^2+^ myeloid cells by secreting CCL2, and these cells bind to NK cells, ultimately blocking tumor immunosurveillance.^496^

In the therapy-induced senescence model, cells undergoing senescence regain proliferative properties through reversibility of senescence, or acquire senescence-associated stemness by reprogramming mechanisms and turn into highly aggressive tumors, which drive tumor recurrence and progression.^485^ Therefore, there may be very complex mechanisms by which aging promotes tumorigenesis.

Senescence has dual roles in tumorigenesis. Whether senescent cells exert tumor-suppressive or tumor-promoting effects depends on numerous factors: the triggers of senescence, the number and persistence of senescent cells, the type and period of tumor tissue, the immune status of the body, and the status of key senescence proteins such as TP53. Despite recent advances in understanding the biology of senescence in cancer, the process of targeting senescence for cancer prevention and treatment still faces many challenges.

## Summary

**Fig. 5:**
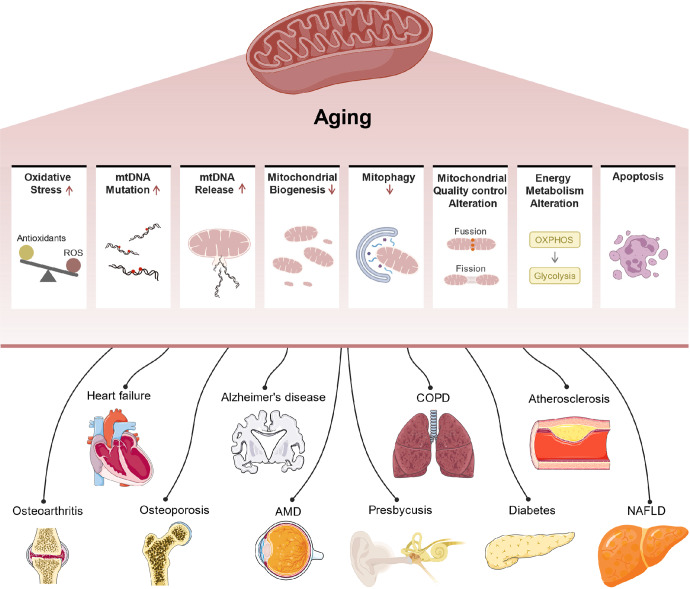
Mitochondrial dysfunction contributes to diverse aging-related diseases. With aging, an increase in ROS production in mitochondria leads to oxidative stress, causing oxidative damage to DNA (especially mtDNA), lipids, and proteins. An increased mtDNA mutation rate causes increased frequencies of errors or mutations in mtDNA-encoded enzyme subunits, resulting in impaired OXPHOS. mtDNA is released into the cytoplasm or outside the cell and participates in SASP secretion by activating cGAS-STING pathways. Decreased mitophagy mediated by the PINK1/parkin ubiquitin pathway results in impaired clearance of damaged mitochondria. Reduced mitochondrial biogenesis mediated by PGC1 and NRF decreases the number of newborn mitochondria. During aging, mitochondria show altered quality control changes, Drp1/FIS1-mediated mitochondrial fission decreases, and MFN/OPA-mediated mitochondrial fusion increases, affecting mitochondrial shape and function. The mitophagy defects and mitochondrial dysfunction trigger Aβ and tau accumulation, leading to synaptic dysfunction and cognitive deficits during AD development. The metabolic transition from OXPHOS to glycolysis leads to altered metabolite generation. Mitochondrial pathway-mediated apoptosis is an important form of cell death. Mitochondrial dysfunction contributes to AD, HF, diabetes, OP, OA, presbycusis, NAFLD, COPD, AMD, and atherosclerosis by inducing oxidative stress, inflammation, apoptosis, and metabolic alterations. (Fig. 5 includes modified templates from Servier Medical Art (http://www.servier.com), licensed under a Creative Commons Attribution 3.0 Unported License.)

**Fig. 6:**
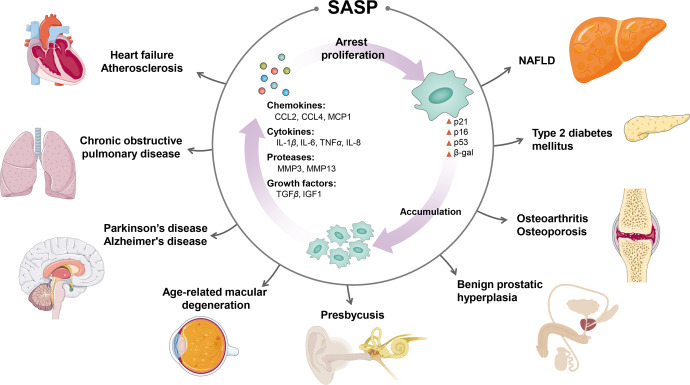
SASP related to various age-related diseases. Senescent cells that have a proinflammatory SASP can cause substantial pathogenic effects, resulting in various aging-related diseases. In the tissue microenvironment, the SASP involves chemokines, cytokines, proteases, and growth factors, which have a range of negative effects on neighboring cells, the surrounding extracellular matrix and other structural components. Senescent cells exhibit increased expression of chemokines, such as CCL2 and MCP1, which promotes the recruitment of monocytes, macrophages and lymphocytes in the vascular endothelium, islets, liver, synovium, and retinas. The accumulation of proinflammatory factors, such as IL-6, IL-1β, TNF-α, and IL-8, exacerbates the pathogenesis of various age-related diseases. Proteases destroy the external BRB and cartilage by inducing matrix degradation in AMD and OA. Growth factors, such as TGF-β and IGF-1, induce the abnormal proliferation of epithelial and stromal cells involved in EMT in BPH. The multifaceted SASP of senescent cells promotes the progression of various diseases and may be a therapeutic target. (Fig. 6 includes modified templates from Servier Medical Art (http://www.servier.com), licensed under a Creative Commons Attribution 3.0 Unported License.)

**Fig. 7:**
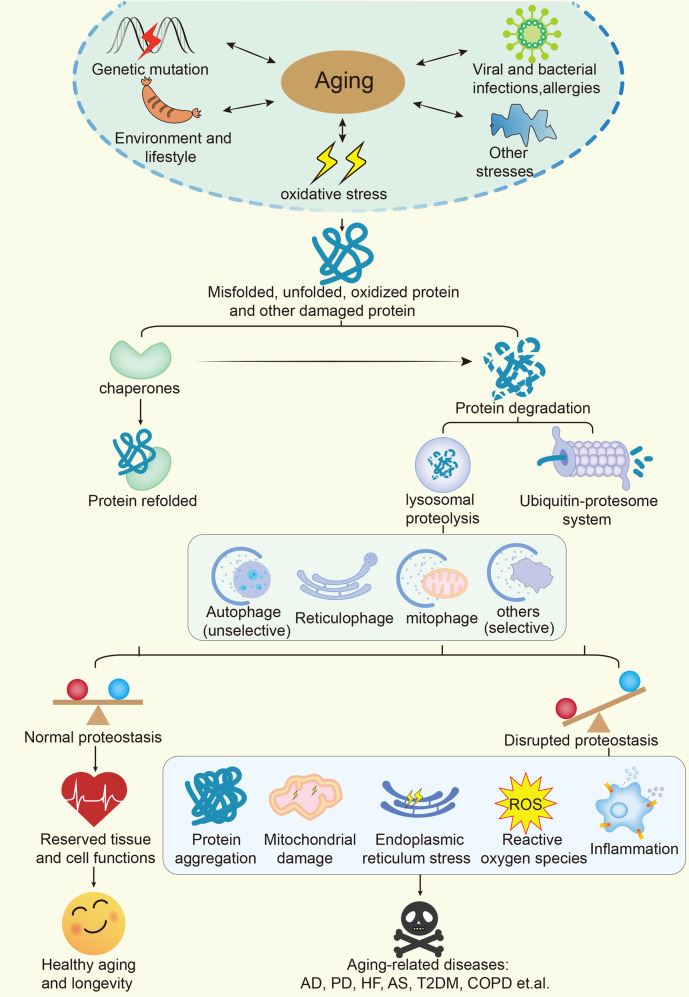
Molecular mechanisms for proteostasis in aging-related diseases. Aging, genetic mutations, environmental and lifestyle insults, and various other stresses cause increases in the amounts of unfolded, misfolded, and oxidized proteins, which lead to activation of the protein degradation system of the UPS and lysosomal proteolysis (including nonselective autophagy and selective autophagy, such as mitophagy and reticulophagy). Chaperones help refold unfolded proteins and assist in the formation of autophagosomes and ubiquitin-proteasomes. Balanced proteostasis leads to healthy aging and longevity. Disrupted proteostasis induces protein aggregation, cellular organelle function loss, increased ROS production and chronic inflammation, which lead to the development of many aging-related diseases

Overall, the existing evidence indicates that the hallmarks of aging are the common drivers of aging-related diseases. However, the diverse aging-related diseases in different organs and systems have their own combinations of molecular hallmarks of aging. Mitochondrial dysfunction (Fig. 5), defective autophagy, loss of proteostasis (Fig. 6), induction of cellular senescence and alteration of intercellular communication by the SASP (Fig. 7) are common causes of various aging-related diseases. Moreover, numerous aging-related diseases are associated with a chronic inflammatory status, which is frequently attributable to the long-term accumulation of senescent cells in various tissues. Other hallmarks of aging, such as genomic instability, epigenetic alteration, telomere attrition, deregulation of nutrient sensing, and dysbiosis of the gut microbiota, are also related to many major aging-related diseases, including cardiovascular diseases, neurodegenerative diseases, metabolic diseases, and chronic respiratory diseases. Therefore, intricate mechanisms underlie different aging-related diseases. Understanding the common and different mechanisms will provide new insights to aid in the development of therapeutic strategies against aging-related diseases.

## Interventions and treatments for aging-related diseases

**Fig. 8:**
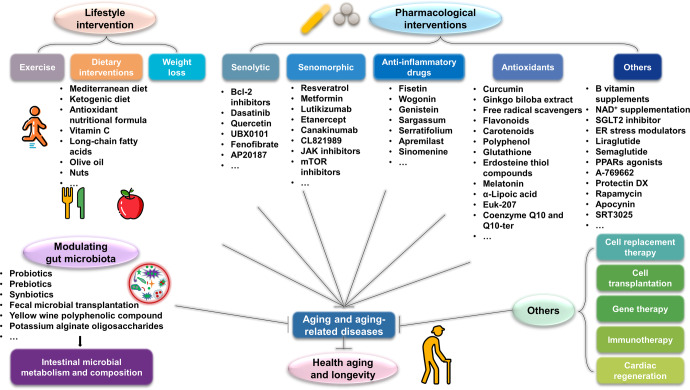
Possible interventions and treatments against aging-related diseases. Proof-of-principle therapeutic strategies used in cell experiments, animal experiments, and clinical trials are shown together. Daily lifestyle changes, such as exercise, dietary interventions, and weight loss, can inhibit aging and reduce the occurrence and development of aging-related diseases, subsequently promoting healthy aging and longevity. Drug therapy is the main strategy targeting aging. Antiaging drugs exert their effects by reducing the number of senescent cells, alleviating the SASP, and exerting anti-inflammatory and antioxidant effects while affecting multiple signaling pathways. Altering the metabolism or composition of the gut microbiota with drugs or through microbiota transplantation can also inhibit aging and aging-related diseases. Moreover, cell replacement therapy, cell transplantation, gene therapy and immunotherapy can be used to promote healthy aging and longevity and to treat aging-related diseases. mTOR mammalian target of rapamycin, NAD^+^ nicotinamide adenine dinucleotide, SGLT2 sodium-glucose cotransporter 2, ER endoplasmic reticulum, BET bromo- and extraterminal

Aging is an inevitable pathophysiological process caused by many factors that lead to a progressive reduction in the ability to resist stress and contribute to a variety of aging-related diseases. Healthy longevity without aging-related diseases has been pursued by humans since ancient times. The prevention and treatment of aging-related diseases is promising but challenging. However, there are many mechanisms of aging, and there are also differences in the development of different aging-related diseases. There are nondrug therapies for aging-related disease prevention and treatment, such as CR, nutrition, exercise, and intestinal microbiota transplantation, as well as drug therapies targeting different aging mechanisms and symptoms of aging-related diseases (Fig. 8). Next, this review will focus on the advances in laboratory and clinical studies on these interventions and treatments in the contexts of different aging-related diseases.

### Alzheimer’s disease (AD)

Before the clinical signs and symptoms of AD appear, lifestyle interventions, including good nutrition and physical exercise, can improve the cognitive status of individuals and attenuate both the development and progression of AD.^497,498^ For example, the Mediterranean diet significantly reduces the risks of cognitive impairment and AD. Some studies have also found that adding olive oil and nuts to one’s diet can improve cognitive function. In addition, long-chain fatty acids, including omega-3 polyunsaturated fatty acids, eicosapentaenoic acid, and docosahexaenoic acid, has been found to be beneficial for cognitive and mental health. These fatty acids can delay aging-related cognitive potential decline as well as AD.^499^ The ketogenic diet is another specific diet studied for its effects on neurodegenerative diseases.^500,501^ In addition, some studies have shown that physical excise may also be a lifestyle intervention that can prevent AD in elderly people.^501^

In terms of drug targets, neurodegenerative diseases share common features of protein aggregation, such as neurofibrillary tangles and Lewy bodies. A common strategy for AD treatment is to clear Aβ through the application of the amyloid cascade hypothesis.^502^ The expression of Aβ is mainly reduced via immunotherapy or by inhibiting γ- and β-secretase.^503^ The aggregation of phosphorylated tau proteins in neurons leads to neuronal damage and AD; therefore, it is considered another potential drug target for AD. However, the long-term effects of these drugs still need to be verified, and results have differed among the existing clinical trials.

In recent years, there has been an increasing emphasis on strategies involving nonamyloid targets for the treatment of AD, including therapeutic approaches to combat inflammation and oxidative stress; to provide synaptic and neuronal protection; to affect vascular factors; to provide mitochondrial protection; and to intervene at the epigenetic level. There has also been an increase in research on “drug repurposing”. Two typical examples of repurposed drugs are escitalopram and metformin.^504–506^ Cognitive impairment caused by elevated homocysteine concentrations has been reported to be inhibited following the administration of B vitamin supplements and may be particularly beneficial in individuals with one or more conditions.^507,508^ Aducanumab is an antiamyloid monoclonal antibody and was the first disease-modifying therapy approved for AD. Several clinical studies on the use of aducanumab for mild cognitive impairment and mild AD in patients started in 2021. The EMERGE trial showed that high-dose aducanumab can slow the progression of clinical cognitive impairment. However, another clinical trial, ENGAGE, did not find a significant protective effect. Therefore, this clinical trial evidence is contradictory, and more phase III studies are needed to determine the efficacy of aducanumab in the future.^509–511^

At present, cholinesterase inhibitors, such as tacrine and donepezil, are primarily used clinically to relieve the symptoms of AD. However, with increasing age and the aggravation of symptoms, the number of active neurons decreases, which makes it difficult for drugs to effectively treat AD. The main currently approved drugs are cholinesterase inhibitors (acetylcholinesterase inhibitors) and N-methyl-D-aspartate (NMDA) receptor antagonists. Acetylcholinesterase inhibitors generally reduce the hydrolysis of acetylcholine released from presynaptic neurons into the synaptic cleft by inhibiting acetylcholinesterase in the synaptic cleft. These effects enhance the stimulation of cholinergic receptors and improve cognitive function in patients with mild to moderate disease.^512,513^ The representative NMDA receptor antagonist is memantine, which can antagonize NMDA receptors and regulate glutamate activity for the treatment of patients with advanced disease.^514^ Sodium oligomannate and aducanumab are indicated for the treatment of moderate stages of AD.^515^ Although these treatments can alleviate cognitive impairment and improve quality of life in patients with mild to moderate AD, clinical evidence in the past 20 years has demonstrated that they have no significant effect on disease onset or progression.^516,517^

A number of researchers have proposed mechanistic links among oxidative stress, inflammation, and neurodegeneration; therefore, the use of natural plant components and dietary antioxidants to prevent neuronal damage may be a therapeutic approach to reduce AD risk.^518^ Examples include drugs targeting Aβ and tau, natural anti-inflammatory antioxidants (such as the polyphenol derivatives curcumin, *Ginkgo biloba* extract, etc.), free radical scavengers, nerve growth factors, and anti-infective treatments. In traditional Chinese medicine, many natural herbs have been indicated to be effective alternative treatments for AD, such as Astragalus, Artemisia, Ginseng, *Ginkgo polygonatum*, Chuanxiong, and *Lycium barbarum*.^519,520^

Decreased removal of dysfunctional mitochondria is an important mechanism of AD. The development of strategies to treat this impairment of mitophagy would benefit from the screening and identification of new mitophagy regulators.^521,522^ Recently, a research team used a combination of unsupervised machine learning (including vector representation of molecular structure, pharmacophore fingerprints and conformational fingerprints) and cross-species methods to screen and validate new mitophagy-inducing natural compounds. They found two effective compounds, kaempferol and rhapontigenin, that increased glutamatergic and cholinergic neuron survival and function, eliminated Aβ and tau lesions, and improved memory in animals.^523^

Recent studies have found that gut microbes are key regulators of many diseases and that gut microbes are involved in neurodegeneration through the gut microbiota–brain axis. This finding supports the possibility of new microbiota-based therapeutic options. Although many studies have found a possible relationship between the gut microbiota and AD pathogenesis, the inconsistency and reproducibility of existing clinical results need to be addressed before clinical application.^196^

### Parkinson’s disease (PD)

There is increasing evidence that the Mediterranean diet, which includes high amounts of fruits, vegetables, whole grains, fish, and unsaturated fatty acids, reduces the incidence of PD.^524^ Many antioxidants, such as flavonoids, carotenoids, quercetin, curcumin, and resveratrol, have been shown to reduce the risk of PD.^525,526^ NAD^+^ supplementation may delay the onset of premature aging and aging-related neurodegeneration.^527^ Some traditional Chinese herbal medicines also inhibit the progression of PD, such as dogwood, ginseng, Pueraria, and skullcap.^528^ Microglia are activated due to aging, exogenous or endogenous infection, oxidative stress, and genetic factors, which may lead to neuroinflammation and neurodegeneration. The prevention of microglial activation represents a potential therapeutic strategy. With the development of science and technology, cell transplantation and gene therapy have brought good prospects to the treatment of PD. However, both are in the experimental stage and have not been used in clinical practice. Notably, transplantation of human induced pluripotent stem cells into primate dopaminergic neurons has been found to lead to the formation of dense neurites in the striatum.^529,530^

Nondrug treatments for PD include surgical treatments that can relieve the clinical symptoms, such as deep brain stimulation and rehabilitation and exercise therapy.^531^ At present, the drugs for the treatment of PD are divided into two main categories: anticholinergic drugs (such as Antan) and drugs related to the action of dopamine in the brain.^532,533^ To improve PD pathology, attempts have been made to reduce α-syn expression. Targeting specific posttranslationally modified forms of α-syn, such as the phosphorylated, nitrated, oxidized or truncated forms, may also be a strategy for PD therapy.^534^ In terms of targeting neuroinflammation, aspirin and ibuprofen are associated with a 13% lower risk of PD development. Masitinib is a novel tyrosine kinase inhibitor that targets the proliferation and activation of mast cells and microglia, reducing the risk of motor nerve injury.^535,536^

Gastrointestinal dysfunction is also an important pathogenic mechanism during the development of PD. In PD, a highly complex relationship between the gut microbiota and the brain, including the vagus nerve and α-Syn in the enteric nervous system, alters gut permeability and inflammation. Additionally, it causes changes in gut microbes and their metabolic activity. However, the results from studies on the link between exposure to certain types of oral antibiotics and an increased risk of PD are inconsistent and still controversial. Moreover, there are also differences in the results of existing animal experiments and human experiments regarding the therapeutic efficacy of FMT.^537^

### Heart failure (HF)

Aging can lead to decreased cardiac function and HF. Antiaging strategies can reduce the progression of HF. For example, drugs that delay cellular senescence and adoption of healthy lifestyles (smoking cessation, physical exercise,^538^ dietary interventions, and weight control) could be effective.^539^ These interventions can be presented directly to the patient along with education and lifestyle guidance, but low adherence hinders their efficacy. However, CR could exert an extensive and unfavorable influence on aging-associated diseases such as nervous system diseases, cardiac diseases and cancer. Additionally, recommendation of one specific diet over another is difficult because of the limited available evidence.^540^ Regarding drugs, treatment with the sodium-glucose cotransporter 2 inhibitor dapagliflozin for 12 weeks has been found to significantly improve cardiac function and exercise capacity in patients with HF with preserved ejection fraction. This treatment improves the quality of life and is well tolerated in patients with chronic HF with preserved ejection fraction.^541^ Navitoclax is a Bcl-2 inhibitor that targets cellular senescence and activates apoptosis in senescent cardiomyocytes. This inhibitor can improve left ventricular systolic function and conduction velocity, and suppress myocardial fibrosis and cardiac hypertrophy in mice with Ang II-induced HF.^542^

Cycloastragenol, a major natural compound in *Astragalus membranaceus*, has been shown to have multiple pharmacological effects, such as antiaging, anti-inflammatory and antifibrotic effects. Cycloastragenol is also the only telomerase activator discovered to date that can delay telomere shortening by inducing telomerase expression.^543^ In rats, cycloastragenol can ameliorate cardiac insufficiency and cardiac remodeling by increasing cardiomyocyte autophagy and inhibiting the expression of matrix metalloproteinase 2 (MMP-2) and MMP-9, indicating that cycloastragenol may be a therapeutic candidate for HF.^544^ Moreover, the dietary safety of cycloastragenol is very good, and its subchronic toxicity and genotoxicity have not been found.^545^ In spontaneously hypertensive rats, *Eriobotrya japonica* leaf extract has been found to effectively reduce cardiomyocyte apoptosis and myocardial fibrosis, and improve cardiac function.^546^ Furthermore, Alpinate Oxyphyllae Fructus has antiapoptotic and prosurvival effects in ROS-induced aging hearts.^547^ *Aconitum carmichaelii* Debeaux has various biological activities, such as anti-inflammatory, analgesia, antiaging and energy metabolism–altering effects, and is currently used for the treatment of cardiovascular diseases such as chronic HF and coronary heart disease.^548^

Cell replacement therapy is a new strategy for HF that is aimed at improving function by replacing dysfunctional cells with sufficient differentiated human stem cells. Li, J. et al. reported that stem cell antigen 1 (Sca1)^+^ cells isolated from the bone marrow of young mice were engrafted into aged mice and could inhibit aging and promote the regeneration of cardiac endothelial cells.^549^ Cell replacement-based therapeutic strategies are viable for designing therapies to enhance the repair and regeneration of injured hearts in humans. Moreover, MSC-based cardiac regenerative treatment may be a novel therapeutic approach. However, the potential cytotoxicity to host cells should be considered when choosing MSC-based cell interventions. These strategies to enhance cardiac cells may further stimulate paracrine effects, and this needs to be taken into account in cardiac regeneration medicine. Currently, there are multiple strategies for cardiac regeneration under active development, each with its own advantages and challenges. In the future, new developments will be necessary to achieve cardiac regeneration. For example, stimulating endogenous cardiac regeneration by mobilizing and modulating resident stem cells is actually the targeting step in most current strategies for cardiac regeneration. Future research and clinical application should also aim to enhance the regeneration capacity of endogenous resident cardiac endothelial cells to promote therapeutic neovasculogenesis in the injured heart. However, both intrinsic and extrinsic regulators need to be taken into account when designing therapeutic strategies to enhance the regeneration of the injured heart. For example, the immunological activity of cells can engage in complex interactions with resident heart cells and the extracellular matrix of tissue, ultimately leading to cell death in the heart.

Aging-induced alterations in gut microbial composition and metabolism are associated with the development of HF. Since the launch of the Human Microbiome Project, intestinal microecology research has developed rapidly, providing new directions for studying the mechanisms of drug action in HF. With regard to deregulated nutrient sensing, studies have shown that interventions affecting the gut microbiota or its related enzymes can regulate intestinal microbial metabolism and reduce circulating trimethylamine oxide (TMAO) levels. Thus, they can treat HF and improve outcomes in HF patients.^550^ In addition, the yellow wine polyphenolic compound prevents doxorubicin-induced HF by regulating the gut microbiota composition and metabolic functions. These findings support the use of dietary polyphenols for the treatment of HF through microbiota modulation.^551^ Furthermore, potassium alginate oligosaccharides alter the composition of the gut microbiota to increase microbial diversity, potentially preventing the development of hypertension and HF in spontaneously hypertensive rats.^552^ Therefore, exploring therapeutic strategies targeting the gut microbiota may enrich the therapeutic concepts of HF and improve therapeutic efficacy against this disease.

### Atherosclerosis

Patients with atherosclerosis should change their lifestyle, control their total intake of calories, and reduce the proportion of fat, especially saturated fat, in their total caloric intake.^553^ Unlike other interventions, lifestyle adjustments have few side effects. In many cases, diet is the main driver, because dietary interventions can reduce ROS production, reduce nuclear DNA and mtDNA damage accumulation, and maintain mitochondrial homeostasis. Over the past few decades, epidemiological, clinical andbasic studies have demonstrated that dietary interventions are key strategies to inhibit aging, promote health and prevent atherosclerosis.^554^ In addition, the combination of a low-fat diet with drug therapy may yield enhanced therapeutic effects.

The Mediterranean diet can prevent atherosclerosis by interfering with multiple signaling pathways that promote the development of the disease. The Mediterranean diet has beneficial effects on blood lipids, lipoprotein particles, inflammation, oxidative stress and the expression of proatherosclerotic genes.^555^ This diet is rich in fruits, vegetables, red wine and olive oil, which contains flavones, polyphenols, and stilbenes that may activate SIRT1. Therefore, this diet can reduce the incidence of multiple aging-related diseases, such as cardiovascular disease, neurodegenerative disease and metabolic disease.^556^ For example, the Mediterranean diet may improve the function of circulating EPCs in elderly individuals, thereby improving endothelial function in cardiovascular disease.^557^

Senolytics are standard antiaging treatments that are designed to identify and target senescent cells. Common drugs in this class include dasatinib and quercetin. In ApoE^−/−^ mice, quercetin increases SIRT1 expression and reduces lipid deposition.^558^ Combination treatment with dasatinib and quercetin reduces aging markers, improves vasomotor function and reduces aortic calcification in hypercholesterolemic mice.^559^ However, the oral bioavailability of quercetin is relatively poor. Moreover, the SASP of senescent cells promotes senescence of the secreting cells and other cells. Therefore, preventing the secretory behavior of senescent cells and eliminating the deleterious effects of intercellular communication can also combat cellular senescence. This idea has resulted in the development of novel antiaging drugs called senomorphics, which are represented by metformin. Metformin is commonly used to treat diabetes and affects longevity through AMPK activation. In ApoE^-/-^ mice, metformin inhibits endothelial cell senescence and atherosclerosis.^560^ In addition, studies have shown that adding a high dose (1%) of metformin to the diet reduces the lifespan of mice.^561^ However, metformin has obvious side effects, such as abdominal distension and diarrhea.^158^

Traditional Chinese medicines such as Bazi Bushen have been shown to regulate aging and treat atherosclerosis through antilipid, anti-inflammatory and antiapoptotic mechanisms.^562^ Berberine (BBR) is an alkaloid derived from plants with anti-inflammatory and lipid-regulatory properties, making it a potential therapeutic candidate for atherosclerosis.^563^ BBR also promotes autophagy in macrophages by activating SIRT1-induced deacetylation of transcription factor EB (TFEB), thereby inhibiting atherosclerosis.^564^ In addition, BBR can downregulate the choline-TMA-TMAO metabolic pathway in intestinal bacteria with vitamin-like effects and treat atherosclerosis.^565^

### Type 2 diabetes mellitus (T2DM)

Lifestyle intervention is an effective and feasible method for reducing diabetes risk.^566^ For example, weight loss resulting from dietary and physical activity interventions effectively reduces the incidence of T2DM.^567^ Pancreatic β cell and adipocyte senescence leads to insulin resistance and worsens metabolic homeostasis. Distinctive potent markers and molecular mechanisms of cellular senescence have been found in these two types of cells. Therefore, the senotherapeutic strategies for adipose tissue and β cells are different.^568^ Senolysis can specifically remove senescent cells without harming healthy cells. In a transgenic INK-ATTAC model, B/B homodimerizer treatment specifically kills p16-positive cells. In addition, in 12-week high-fat diet (HFD)-fed mice, B/B homodimerizer treatment improves β cell function and glucose tolerance.^283^ A senolytic drug, ABT263 (a Bcl-2 inhibitor), also improves glucose metabolism and β-cell function and decreases the expression of markers of aging, senescence, and the SASP in an HFD-fed mouse model.^283^ Unfortunately, ABT263 has possible adverse effects; specifically, it is toxic to platelets and induces transient thrombocytopenia and thrombopathy.^569^ Other senolytic drugs, such as ABT-199 (a Bcl-2 inhibitor) and ABT737 (a Bcl-2, Bcl-xL, and Bcl-w inhibitor), can also remove senescent β-cells.^570^ Dasatinib and quercetin have been found to eliminate both p16^high^ and p21^high^ senescent cells in the pancreas and adipose tissues in a T2DM animal model.^571,572^ Unexpectedly, it has been reported that treatment with dasatinib and quercetin worsens liver disease progression in a diethylnitrosamine (DEN)/HFD mouse model, slightly increases histological damage and tumorigenesis, and has no effect on senescent cell removal.^573^

Moreover, human senescent β cells also respond to senolysis, establishing the foundation for translation. These novel findings lay the framework to pursue the senolysis of β cells as a preventive and alleviating strategy for T2DM.^283^

In addition to promoting senolysis, the senomorphic drugs resveratrol and metformin can ameliorate β-cell senescence and improve metabolic balance in animal models.^574,575^ In diabetic individuals, metformin is suggested to improve glucose tolerance and insulin sensitivity by reducing telomere shortening, preventing inflammation and oxidation and shifting the gut macrobiotia composition.^576–579^ However, the use of metformin by older adults can cause deficiencies in folate-related B vitamins, which are associated with reduced cognitive performance.^580^

Additionally, impaired ER homeostasis has been found to induce the misfolding of proinsulin and the pathogenesis of diabetes. Numerous studies have found that administration of the ER stress modulators 4-PBA and TUDCA improves β-cell functions and the secretion of insulin.^581^ PERK inhibitors have also been shown to improve the secretion of insulin.^582^ Modulating the gut microbiota with probiotics, prebiotics, synbiotics and via fecal microbial transplantation is a promising approach to treat diabetes.^287^ The Chinese traditional medicine BBR is a natural alkaloid extracted from *Berberis aristata and Coptis chinensis*. It has been confirmed that BBR has hypoglycemic effects in Chinese participants by modulating the gut microbiota.^583^

### Nonalcoholic fatty liver disease (NAFLD)

Lifestyle modifications, including dietary changes, physical exercise, and weight loss, are the basic treatments for NAFLD. Weight loss is the key goal of lifestyle modifications. Studies have reported that a weight loss of 5% reduces hepatic fat content by approximately 30% and that a weight loss of 7–10% may be sufficient to reduce hepatic inflammation in obese subjects suffering from NAFLD.^584^

Polyphenols are antioxidants that provide beneficial effects for NAFLD patients.^585^ Vitamin C at a dose of 800 mg/day improves hepatic histological pathologies in NASH patients without T2DM.^586^

A meta-analysis has shown that supplementation with probiotics in NAFLD patients can decrease the expression of inflammatory factors in the liver.^587^ Other research has shown that FMT from healthy individuals to NAFLD patients can improve small intestinal permeability, but FMT has only minimal effects on liver dysfunction and metabolic syndrome.^588^

No drug is currently approved for the treatment of NAFLD. However, because the incidence of NAFLD is closely related to T2DM and obesity, various targeted antidiabetic and antiobesity drugs have been evaluated for NAFLD treatment. Metformin, a first-line antidiabetic drug, has been shown to reduce body weight, protect against oxidative stress, preserve mitochondrial function, and inhibit cellular senescence. However, a meta-analysis has reported that although metformin improves liver function and increases insulin sensitivity to some extent, it does not improve NASH-related outcomes.^587^ Liraglutide and semaglutide are two analogs of GLP-1 that have been tested in patients with NAFLD. Liraglutide promotes weight loss and can reduce hepatic steatosis. Additionally, semaglutide reduces hepatic fat accumulation in patients with NASH, but the effect on fibrosis has not been proven.^589^ PPARs agonizts play critical roles in regulating hepatic fatty acid β-oxidation. Additionally, they have been demonstrated to improve insulin sensitivity and to have anti-inflammatory properties. A phase 2 clinical trial has shown that saroglitazar, a double agonist of PPARα/γ, can significantly improve liver function, attenuate insulin resistance, ameliorate dyslipidemia and decrease hepatic steatosis in patients with NAFLD or NASH.^590^ In addition, elafibranor, a PPARα/β/δ agonist, was used in the GOLDEN-505 trial with 247 NASH patients. In this study, elafibranor could not achieve histological resolution in 162 patients with NASH but ameliorated insulin resistance, improved plasma lipid profiles and attenuated hepatic steatosis in a subgroup with significant inflammation.^591^

In addition, several traditional Chinese medicines have been identified as promising candidates for NAFLD treatment. BBR, an active single compound isolated from Rhizoma Coptidis, has been verified to significantly ameliorate hepatic steatosis in NAFLD patients in a randomized clinical trial and to directly regulate the expression of genes related to hepatic lipid metabolism. However, anorexia, upset stomach, diarrhea and constipation are common side effects in patients treated with BBR.^348^ Cordycepin, the major component of the fungus *Cordyceps militaris*, has been reported to protect against hepatic steatosis, inflammation, and fibrosis in mice through stimulation of the AMPK signaling pathway.^592^ Breviscapine, isolated from the traditional Chinese herb *Erigeron breviscapus*, attenuates hepatic lipid accumulation, inflammation, and fibrosis in mice via direct inhibition of TGF-β-activated kinase 1.^593^

### Osteoarthritis (OA)

A senolytic cocktail of dasatinib and quercetin effectively has been found to eliminate senescent cells in joints and to alleviate OA symptoms in an older mouse model.^370,594^ UBX0101, an E3 ubiquitin-protein ligase, specifically degrades p53 by inhibiting the binding of p53 and MDM2. In one mouse model, injection of UBX0101 selectively eliminated senescent cells and reduced proteoglycan loss, which alleviated the cartilage degradation and pain caused by OA.^339^ Fenofibrate, a PPARα agonist, clears SA-β-gal-positive chondrocytes by promoting apoptosis.^595^ Fisetin, a flavonoid drug, alleviates inflammation and prolongs the lifespan of chondrocytes.^596,597^ Navitoclax (ABT263) eliminates p16-positive chondrocytes by promoting apoptosis.^598^

Senomorphics remove the SASP of senescent cells. Thus far, drugs targeting TNF or IL-1, such as lutikizumab (an inhibitor of IL-1α and IL-1β), etanercept (a TNF inhibitor), and canakinumab (an anti-IL-1β antibody), have achieved satisfactory therapeutic results in clinical trials.^599–601^ Specific knockout of MMP13 in chondrocytes alleviates OA-induced meniscal–ligamentous injury. Treatment of mice with CL821989 (a selective inhibitor of MMP13) relieves the symptoms of OA by increasing collagen content and inhibiting chondrocyte death.^602^ Metformin, quercetin, A-769662 and Protectin DX have been found to alleviate OA progression through the AMPK pathway in a mouse OA model.^348,603,604^ Rapamycin has been found to delay articular cartilage degeneration by activating autophagy in a murine model of osteoarthritis.^605^ Rapamycin has many side effects, such as hyperlipidemia, gastrointestinal disorders, and respiratory and urinary infections, but many of these effects are reversible.^606^ In addition, many drugs, such as wogonin, genistein, *Sargassum serratifolium*, apremilast and sinomenine, have been shown to alleviate the progression of OA by reducing inflammation.^607–611^

### Osteoporosis (OP)

Elimination of senescent cells can alleviate the pathogenesis of OP. The INK-ATTAC “suicide” transgene encoding inducible caspase 8 is expressed specifically in senescent cells and specifically kills senescent cells in mice.^284,559,612^ In an old INK-ATTC mouse model, administration of AP20187 has been found to specifically eliminate p16-positive cells. AP20187 treatment reduces senescent bone cells, alleviates trabecular bone loss in the spine and bone cortex loss in the femur and improves bone strength.^370^ However, this is not the approach proposed for application in humans. Senolytics target the senescent cell antiapoptotic pathway and lead to the apoptosis of senescent cells without harming healthy cells.^613,614^ Injection of a senolytic combination (dasatinib and quercetin) into 20-month-old male mice reduces p16 expression and the proportion of senescent bone cells in bone.^613^ Senomorphic drugs, such as JAK inhibitors and mTOR inhibitors, do not kill senescent cells but inhibit the secretion of SASP components.^615^ The JAK inhibitor ruxolitinib reduces the size of the secretome derived from senescent cells.^370,615^ Ruxolitinib treatment reduces the levels of proinflammatory factors and improves spine trabecular bone microarchitecture and bone strength.^370^ It has been reported that other drugs can also inhibit bone loss and improve bone mass and microstructure in animal models, such as resveratrol, apocynin (an inhibitor of NADPH oxidases) and SRT3025 (a Sirt1 activator).^366,616,617^

MSCs are multipotent cells capable of differentiating into osteoblasts, adipocytes, or chondrocytes.^596^ MSC transplantation promises to increase osteoblast differentiation, block osteoclast activation and rebalance bone formation and resorption.^618^ Many ncRNAs and extracellular vesicles transplantation therapies are promising approaches to enhance the therapeutic effects and efficacy of MSCs.^619–624^

Targeted inhibition of cellular senescence is a novel therapeutic strategy for preventing or even reversing age-related OP and concurrently treating a variety of other aging-related diseases. This approach does not focus on the bones but on the basic aging mechanisms of the whole body. If the results of clinical studies are consistent with those of preclinical studies, it may be possible to improve the health of the aging population.

### Chronic obstructive pulmonary disease (COPD)

Senolytics is an antiaging treatment that has attracted much attention recently, and dasatinib and quercetin have been used in clinical trials for idiopathic pulmonary fibrosis.^625^ However, to date, no convincing clinical trials have confirmed that senolytic therapies are effective and safe for COPD.^626^ MSCs have also been used as a potential treatment for COPD.^627,628^ In COPD rats, transplanted MSCs repair damaged tissues through anti-inflammatory effects, inflammation regulation and growth factors released by the MSCs. Thus far, some research teams have tried to implant functional lung tissue formed by lung stem cells to improve gas exchange capacity in COPD, but they have not achieved the desired effect.^629^ Antioxidant therapy is also a current strategy to combat the aging process and may protect against COPD. Studies have shown that atomized glutathione treatment can improve the respiratory function of COPD patients,^630^ and erdosteine thiol compounds have been found to decrease exacerbations and shorten hospital stays.^631^ Resveratrol has anti-inflammatory and antioxidant properties, making it a potentially useful treatment for aging-related COPD.^632,633^ In patients with COPD and diabetes, metformin treatment can reduce COPD-specific emergency treatment and hospitalization rates.^634^ However, the side effects of metformin are had been apparent in some other clinical trials, such as diarrhea and bloating.^635^ Furthermore, melatonin treatment improves dyspnea in COPD patients.^636^

Traditional Chinese medicines are also used for the treatment or adjuvant treatment of COPD. In a preclinical study, curcumin reduced pneumonitis manifestations in COPD patients by suppressing apoptotic signaling via regulation of oxidative stress.^637^

### Benign prostatic hyperplasia (BPH)

Dietary prophylaxis can antagonize the hormonal regulation of BPH and reduce the development of BPH. The useful dietary compounds include polyphenols, isoflavones, and flavonoids that are present in fruits, broccoli, and green tea.^638^ Androgen-induced prostate cell growth can also be inhibited using drugs that modulate cellular metabolism. For example, the widely used antidiabetic drug metformin has hypoglycemic effects and antiproliferative and proapoptotic properties. Metformin inhibits the effects of testosterone on estrogen receptor alpha upregulation and estrogen receptor beta downregulation during BPH. Although metformin does not affect 5α-reductase, it decreases the mRNA levels of IGF-I and its receptor. IGF regulates cell proliferation and apoptosis through a complex system that is dependent on receptor and binding protein expression.^639^

In recent years, drug treatments for BPH have included α1-adrenoceptor blockers (α-blockers), 5α-reductase inhibitors, phosphodiesterase inhibitors, and gonadotropin-releasing hormone drugs. Treatment of BPH with the 5α-reductase type II inhibitor finasteride is a standard clinical procedure. Concomitant administration of dutasteride and testosterone inhibits the growth of BPH-1 cells.^637^ However, 5α-reductase type II inhibitors can cause side effects such as sexual dysfunction and allergic reactions, which should be monitored during drug application. Vanillic acid has been shown to inhibit AR, estrogen receptor alpha and steroid receptor coactivator-1.^640^ Treatment with vanillic acid (a known anti-inflammatory agent) results in decreased prostate weight and increased epithelial thickness. The neuropeptide 5-HT also attenuates AR expression, thereby preventing prostate branching.

Transurethral resection of the prostate is a standard surgical procedure and remains the most clinically effective long-term treatment. As stated in the BPH/lower urinary tract symptoms (LUTS) guidelines, the surgical approach should be individualized based on surgical risk (anesthesia and bleeding), prostate volume, and patient preference (preservation of sexual function).^641^

### Aging-related macular degeneration (AMD)

The Bcl-2-inhibitory drugs UBX1325 and UBX1967 show promise for the treatment of AMD by promoting apoptosis in senescent cells. In addition, small compounds targeting prosurvival pathways, such as inhibitors of bromo- and extraterminal domain proteins, show prospective therapeutic value by ameliorating RPE cell loss in vitro and protecting ganglion cells in vivo.^642,643^

Several induced pluripotent stem cell (iPSC)-derived RPE cell therapies are in the clinical trial stage and are promising strategies for improving AMD treatment in the near future. Transplantation of RPE cell monolayers or biological patches consisting of RPE monolayers derived from human embryonic stem cells into elderly patients with dry AMD results in improved corrected visual acuity.^644,645^ In addition, treating AMD by transplanting RPE cells derived from skin fibroblast-generated pluripotent stem cells (iPSCs) improves the VFQ-25 score.^646^

Many clinical trials have analyzed the role of antioxidative stress in AMD therapy and have shown a certain beneficial effect on the clinical presentation of AMD. For example, antioxidant nutrient formulas;^647^ the Mediterranean diet;^648^ and some traditional medicines, such as resveratrol,^649^ coenzyme Q1050 and melatonin,^650^ have been used.

The ameliorative effects of traditional Chinese medicine formulas on AMD have been observed among small samples. Several compounds with potential therapeutic effects in Chinese medicines that reduce oxidation, inflammation and apoptosis have been identified by in vitro experiments. However, there is still a lack of multicenter, large-sample, randomized controlled studies to evaluate the effects of traditional Chinese medicines in the treatment of AMD.^651^

### Presbycusis

Aspirin has anti-inflammatory and antioxidant properties that target the senescence-like phenotype and proinflammatory cytokine secretion. A randomized double-blind clinical trial is currently being conducted to evaluate the potential efficacy of low-dose aspirin in the treatment of presbycusis,^652^.^653^ However, aspirin has been reported to increase the risk of gastrointestinal bleeding in healthy elderly individuals.^654^

Stem cell therapy is also used in the treatment of presbycusis. Intracerebral neural stem cell transplantation in C57BL/6 J mice with presbycusis slightly restores auditory function.^655^ In animal models of GABAergic ouabain-induced deafness, such as gerbils, transplantation of otic neural progenitor cells improves hearing,^656,657^ but exogenous cell transplantation in the inner ear of humans is still a challenge.^658^

Antioxidants are also used in the treatment of ARHL. The enzyme cofactor α-lipoic acid,^659^ melatonin,^660^ antioxidant extracts from *Ginkgo biloba* leaves^661^ (EGb761), the synthetic superoxide dismutase/catalase simulant euk-207,^478^ coenzyme Q10, and the coenzyme Q10 analog coenzyme Q10-ter^457^ exert certain preventative effects against ARHL by improving mitochondrial function and ameliorating oxidative stress in animal models. A protective effect of dietary vitamin C intake against ARHL has been observed in elderly patients,^662^ but a protective effect against ARHL was not observed in a double-blind randomized clinical trial on EGb761.^663^

### Cancer

Inducing cancer cell senescence is one of antitumor method by inhibiting cancer cell proliferation.^664^ SASP released by senescent cancer cells recruit immune cells and suppress the growth of adjacent tumor cells. However, chronic and long-term accumulation of SASP will promote tumor growth and metastasis.^665^ Therefore, an ideal method to eliminate tumor cells can include two steps: senescence-inducing therapy and senolytic therapy.^486^

There are many factors that can induce senescence of tumor cells, such as chemotherapy,^666^ CDK upregulation,^667^ telomere attrition,^668^ epigenetic modulation,^669^ and oncogenes induction.^670^ Chemotherapy drugs can trigger senescence in many types of cancer, such as topoisomerase I and II inhibitors (Doxorubicin, daunorubicin, mitoxantrone etoposide and camptothecin),^671–674^ Platinum-based compounds (cisplatin, carboplatin, oxaliplatin),^675–679^ alkylating agents (temozolomide, dacarbazine and busulfan),^680^ and microtubule inhibitors (paclitaxel, docetaxel and vinca alkaloids).^681,682^ Cell cycles of tumor cells can be arrested by using CDK inhibitors to inhibit tumor proliferation. For example, CDK4/6 inhibitors (Palbociclib, abemaciclib and ribociclib),^683^ CDK2/4/6 inhibitor (PF-06873600),^684^ CDK7 inhibitors (ICEC0942, SY-1365, SY-5609 and LY3405105),^685^ and CDK12 inhibitor (CDK12-IN-3)^686^ are potently used in treatment of various tumors. Telomerase inhibitions (BIBR15 and GRN163L) can induce senescence of tumor cells.^687–689^ DNA methyltransferases inhibitor (Decitabine)^690^ and histone deacetylase inhibitors (GCN5, p300/CBP, PCAF, and Tip 60)^669^ induce senescence in tumor cells via Epigenetic modulation.

The SASP factors derived from senescent tumor cells can mediate senescence of neighbor cells. Senescent tumor cells secrete the inflammatory cytokine IL-1α, IL-6, and IL-8. Proinflammatory cytokines and infiltrating immune cells can eliminate tumor cells and suppress tumor progression. However, persistently suffering SASP factors may promote tumor growth and metastasis. Senolytic agents can selectively eliminate senescent cells and minimize side effect of senescence-inducing therapy.^486^ Antiapoptotic BCL-2 family proteins (BCL-2, BCL-XL, and BCL-W) are therapeutic targets for eliminating senescent tumor cells. Navitoclax (ABT263 and ABT737) specifically eliminates various senescent cells via inhibiting BCL-2 family proteins and promoting cell apoptosis.^598,691,692^ And Dasatinib, quercetin, and Fisetin eliminate senescent cells via inhibiting PI3K-AKT pathway.^693–695^

Due to the heterogeneity of tumors, there is no drug that can induce senescence of all tumor cells. In addition, in existing treatments that induce tumor cell senescence, it is difficult to accurately assess the effect of drug-induced tumor cell senescence with current technology. Second, senescence-inducing drugs do not only induce tumor cell senescence but also cause the senescence of normal cells. In addition, although instantly accumulated SASP can promote tumor cell senescence and suppress tumors, sustained SASP can promote cancer progression, which is also an issue to be addressed. Therefore, the search for tumor-specific targeted senescence-inducing drugs or therapeutic approaches in senescence therapy is an important research direction for future tumor therapy.

## Summary

Antiaging interventions and treatments for aging-related diseases face great challenges. If the function of aging-related signaling pathways in physiological processes is compromised, patients receiving antiaging therapy may experience side effects. Over the past decade, antiaging drugs have continually emerged and many drugs have entered the clinical research stage (Table 1). There are increasing cases of toxicity and side effects caused by high-dose and prolonged use of antiaging drugs. For example, metformin, known to lower blood sugar and delay aging, is the most widely used oral hypoglycemic drug for T2DM worldwide and can also reduce the incidence of human aging-related diseases such as neurodegenerative diseases. However, it also has many side effects, some of which may have severe consequences. Therefore, before metformin becomes the mainstay of antiaging therapy, a better understanding of the mechanisms underlying its beneficial effects on aging is required.^635^ The adverse effects of antiaging therapy need to be overcome. In addition, combining antiaging therapies with other established strategies for aging-related diseases may yield better therapeutic outcomes than individual strategies. Future opportunities and challenges will involve determining the optimal combination of synergistic treatment regimens for each patient.

**Table 1:** The clinical trials for aging-related disease treatments

Diseases	Treatments	Effects	Trials	References
AD	Dietary changes in the FINGE	Adherence to healthy diet improves global recognition. Dietary improvement was associated with beneficial changes in executive function.	NCT01041989	^497,498^
AD	Lifestyle Interventions	the FINGER trial showed that a multidomain lifestyle intervention can benefit cognition in elderly people with an elevated risk of dementia.	NCT00672685	^499^
AD	Metformin	T2DM was not related to cognition, but higher glycated hemoglobin at year 8 was related to worse cognition after confounder adjustment. Cumulative metformin exposure was not related to cognition.	NCT00004992 NCT00038727	^505^
AD	Metformin	Metformin was associated with improved executive functioning, learning/memory and attention. No significant changes in cerebral blood flow were observed.	NCT01965756	^506^
AD	B Vitamins	The accelerated rate of brain atrophy in elderly with mild cognitive impairment can be slowed by treatment with homocysteine-lowering B vitamins.	ISRCTN94410159	^580^
AD	Aducanumab (antiamyloid)	Aducanumab’s efficacy for the cognitive dysfunction in AD can’t be proven by clinical trials with divergent outcomes. The ENGAGE trial showed no benefit while the high-dose. EMERGE trial initially also showed no benefit but with longer follow-up there was a significant positive benefit.	NCT02484547 (EMERGE) NCT02477800 (ENGAGE)	^510,511^
HF	Dapagliflozin (SGLT2 inhibitor)	12 weeks of dapagliflozin treatment significantly improved patient-reported symptoms, physical limitations and exercise function and was well tolerated in chronic HFpEF.	NCT03030235	^541^
T2DM	BBR	Berberine has hypoglycemic effects in Chinese participants with T2DM by modulating the gut microbiota.	NCT02861261	^583^
NAFLD	Distinct lifestyle Interventions	Lifestyle interventions promotes weight loss and reduces hepatic reduces hepatic fat content.	NCT01530724	^584^
NAFLD	Fecal microbiota transplantation (FMT)	FMT did not improve ischemia reperfusion or hepatic lipid.	NCT02496390	^588^
NASH	Liraglutide (GLP-1analogues)	Nine (39%) of 23 patients who received liraglutide and underwent end-of-treatment liver biopsy had resolution of definite NASH.	NCT01237119	^589^
NAFLD/ NASH	Saroglitazar (PPARα/γ agonist)	Saroglitazar can improve liver function, attenuate ischemia reperfusion, ameliorate dyslipidemia and decrease hepatic steatosis in patients with NAFLD or NASH.	NCT03061721	^590^
NASH	Elafibranor (PPARα/δ agonist)	Elafibranor could not achieve histological resolution in 162/247 patients with NASH, but ameliorated ischemia reperfusion, improved plasma lipid profiles and attenuated hepatic steatosis in a subgroup with significant inflammation.	NCT01694849	^591^
NAFLD	BBR	BBR ameliorates NAFLD and related metabolic disorders by regulation of hepatic lipid metabolism.	NCT00633282	^348^
OA	Lutikizumab (IL-1α/β inhibitor)	Lutikizumab has limited improvement in the WOMAC pain score in OA patients.	NCT02087904	^599^
OA	Canakinumab (IL-1β inhibitor)	Canakinumab significantly reduces OA related symptoms and replacement rates of total hip and knee during a median follow-up period of 3.7 years.	NCT01327846	^601^
COPD	MSC therapy	MSC therapy ameliorates airway inflammation and stimulates lung tissue repair in patients with COPD.	NCT02645305 NCT01559051	^628^
COPD	Erdosteine	Erdosteine can reduce both the rate and duration of exacerbations in patients with COPD.	NCT0103230	^631^
COPD	Metformin	Metformin decreases the odds of COPD-specific ER visits and hospitalizations in patients with low-complexity COPD.	NCT01247870	^634^
AMD	hESC-derived RPE transplantation	Transplantation of hESC-derived RPE into elderly patients with dry AMD results in improved corrected visual acuity.	NCT01345006 NCT01344993	^644^
AMD	Nutritional Interventions	Nutritional supplementation decreases the prevalence of the early stages of AMD and AMD progression.	NCT00763659	^647^

## Conclusion and future perspectives

For thousands of years, a healthy longevity has been the goals pursued by human beings. With the establishment and improvement of the public health system, human life expectancy has increased significantly, but also faces the challenge of increasing the number of aging-related diseases. Research on aging-related diseases has also become a major focus of scientific research and public health systems. New research methods, new drugs, and new clinical treatments are emerging, offering opportunities for revealing the mechanisms of aging, and the prevention, or treatment of aging-related diseases. The establishment and use of new methodologies such as genomics, proteomics, metabolomics, single-cell transcriptomics, and artificial intelligence applications can directly target aging and aging-related diseases for the study of mechanisms and exploration of prevention and treatment methods.

In the first part of this review, we summarize new developments in the onset of aging at three different levels: the molecular level, the cellular level, and systemic alterations. The molecular mechanisms underlying the onset of aging are summarized from multiple perspectives, at multiple levels and in terms of multi-organ interactions.

Research on the mechanisms of aging is a very active area in academia and a difficult area of research in the biomedical field. The study of aging mechanisms will be extremely important for delaying aging, reducing the occurrence of aging-related diseases, and maintaining a long and healthy life in human body. Especially, in recent years, research on epigenetic regulation, proteostasis, autophagy, cellular senescence, stem cell, has provided us with new directions for aging mechanism study. However, the causes of human aging are multifaceted, and the mechanisms of aging are extremely complex. So far, although a variety of theories on the mechanism of aging have been proposed by academics, all of which have their own experimental basis, but they all have their limitations to explain the complex mechanisms of aging. Therefore, we should adopt a comprehensive and multi-perspective approach when we explain the aging mechanisms. At present, there has been great progress in the research on the molecular mechanisms of aging, and there has been a breakthrough in the understanding of the biological and genetic mechanisms of the aging process, as well as a profound understanding of the pathogenesis of aging-related diseases. However, these findings are still far from being able to delay human aging and reduce the occurrence of aging-related diseases. Therefore, there is still a long way to go in the study of aging mechanisms.

In the second and third section, we summarize the characteristics of the mechanisms of aging-related disease development, and present the latest interventions and therapeutic approaches for aging-related diseases in response to aging. Recently, new research technologies, new drugs, and new clinical treatment methods are emerging, offering new opportunities for revealing the regulation mechanisms of aging and the treatment of aging-related diseases. Although some of the technologies and methods are still in the experimental stage and have not been directly used for the diagnosis and treatment of aging-related diseases, they have good future applications in the future.

Antiaging interventions and treatments for aging-related diseases face great challenges. An important way to achieve healthy aging is through early intervention and prevention. Early lifestyle intervention can promote healthy longevity and reduce aging-related diseases. Through a variety of lifestyle interventions, it is hoped that the aging process can be slowed and the incidence of aging-related diseases may be reduced. In terms of pharmacological interventions and treatments for aging-related diseases, most of the drugs now applied clinically may focus more on symptom relief after the onset of disease and lack therapeutic approaches to address the causes of aging and aging-related diseases. Research on the latest therapeutic approaches, such as stem cell transplantation, elimination of senescent cells, promotion of antiaging factor expression and inhibition of pro-aging factor expression, and tissue or organ regeneration, provides new directions for treatments of aging-related diseases. In addition, there have been significant technological developments, mainly through gene therapy, nanomaterial drug carriers, therapeutic antibodies or small molecule drugs, which have also contributed to advances in the treatment of aging-related diseases. Some of these methods and technologies have been applied in the clinic, and some are undergoing model animal studies and small-scale clinical studies. The application of these state-of-the-art technological approaches and new targeted drugs will facilitate the treatment and clinical application of aging-related diseases.

Interestingly, various alternative medicine approaches including traditional Chinese medicine treatment, acupuncture, dietary supplementation, etc., have also shown certain efficacy in the antiaging and the treatment of aging-related diseases. However, some of these methods still lack systematic mechanistic studies and clinical evidence-based medical evaluations, which may be directions for future research. Despite many advances in the study of human aging-related mechanisms and interventions, many contributing factors remain unclear. Therefore, the effects of these interventions need to be validated through long-term studies. More basic and in-depth clinical research needs to be conducted to discover new methods to fight aging and aging-related diseases. Large-scale evidence-based medical studies are also needed to verify the feasibility and safety of these methods in order to avoid adverse reactions and maximize the benefit to elderly individuals.

The goal of aging medicine is gradually changing from disease treatment to prevention of the occurrence and development of aging-related diseases. In other words, geriatric medicine is moving away from focusing on postdisease treatment to targeting aging-related chronic disease risk factors for preintervention. Research on the mechanisms of aging and on intervention measures and methods has an important role in improving human health and prolonging lifespan. Due to the aging of the global population, antiaging and healthy aging pursuits are undoubtedly important tasks for public health organizations, scientific research departments and drug research and development departments. Although there are many challenges to the research of aging and aging-related diseases and many questions still need to be addressed, promoting healthy aging has important and far-reaching socioeconomic and public health implications. With the emergence of new technologies and methods of modern biology, and the development and utilization of new drug discovery methods, research on aging mechanisms will further facilitate the prevention, diagnosis, and treatment of aging-related diseases, thus promoting healthy longevity for humans.
